# Reduced Expression of Autophagy Markers and Expansion of Myeloid-Derived Suppressor Cells Correlate With Poor T Cell Response in Severe COVID-19 Patients

**DOI:** 10.3389/fimmu.2021.614599

**Published:** 2021-02-22

**Authors:** Sergej Tomić, Jelena Đokić, Dejan Stevanović, Nataša Ilić, Alisa Gruden-Movsesijan, Miroslav Dinić, Dušan Radojević, Marina Bekić, Nebojša Mitrović, Ratko Tomašević, Dragan Mikić, Dragoš Stojanović, Miodrag Čolić

**Affiliations:** ^1^Department for Immunology and Immunoparasitology, Institute for the Application of Nuclear Energy, University of Belgrade, Belgrade, Serbia; ^2^Laboratory for Molecular Microbiology, Institute of Molecular Genetics and Genetic Engineering, University of Belgrade, Belgrade, Serbia; ^3^Clinical Hospital Center Zemun, Faculty of Medicine, University of Belgrade, Belgrade, Serbia; ^4^Clinics for Infectious and Tropical Diseases, Military Medical Academy, Belgrade, Serbia; ^5^Department for Medical Sciences, Serbian Academy of Sciences and Arts, Belgrade, Serbia

**Keywords:** COVID-19, myeloid-derived suppressor cells, autophagy, regulatory lymphocytes, cytokines

## Abstract

Widespread coronavirus disease (COVID)-19 is causing pneumonia, respiratory and multiorgan failure in susceptible individuals. Dysregulated immune response marks severe COVID-19, but the immunological mechanisms driving COVID-19 pathogenesis are still largely unknown, which is hampering the development of efficient treatments. Here we analyzed ~140 parameters of cellular and humoral immune response in peripheral blood of 41 COVID-19 patients and 16 age/gender-matched healthy donors by flow-cytometry, quantitative PCR, western blot and ELISA, followed by integrated correlation analyses with ~30 common clinical and laboratory parameters. We found that lymphocytopenia in severe COVID-19 patients (n=20) strongly affects T, NK and NKT cells, but not B cells and antibody production. Unlike increased activation of ICOS-1+ CD4+ T cells in mild COVID-19 patients (n=21), T cells in severe patients showed impaired activation, low IFN-γ production and high functional exhaustion, which correlated with significantly down-regulated HLA-DR expression in monocytes, dendritic cells and B cells. The latter phenomenon was followed by lower interferon responsive factor (IRF)-8 and autophagy-related genes expressions, and the expansion of myeloid derived suppressor cells (MDSC). Intriguingly, PD-L1-, ILT-3-, and IDO-1-expressing monocytic MDSC were the dominant producers of IL-6 and IL-10, which correlated with the increased inflammation and accumulation of regulatory B and T cell subsets in severe COVID-19 patients. Overall, down-regulated IRF-8 and autophagy-related genes expression, and the expansion of MDSC subsets could play critical roles in dysregulating T cell response in COVID-19, which could have large implications in diagnostics and design of novel therapeutics for this disease.

## Introduction

Coronavirus Induced Disease 2019 (COVID-19) started in Wuhan, Hubei, China in December 2019, has infected more than 36 million people in 10 months, with more than 1 million deaths worldwide. The patients infected with Severe Acute Respiratory Syndrome Corona Virus (SARS-CoV)-2 develop various symptoms, ranging from asymptomatic to mild, moderate, severe, and critical, the latter three requiring hospitalization and intensive care monitoring, including mechanical ventilation. Critical COVID-19 pathology is usually followed by complications including respiratory failure, acute respiratory distress syndrome (ARDS), sepsis, thromboembolism and multiorgan failure (1). A huge capacity of SARS-CoV-2 to infect different organs and tissues is enabled *via* its spike (S) protein which interreacts with widely expressed angiotensin-converting enzyme 2 (ACE2) and the serine protease TMPRSS2 (2, 3). Besides direct pathogenic effects of the virus, COVID-19 pathogenesis seems to be caused by overactivated immune response, characterized by cytokine release syndrome (4), lymphocytopenia and neutrophilia (5). However, immunological mechanisms involved in immune dysregulation and progression of COVID-19, and thereby key potential targets for therapy, are still largely unresolved.

Both adaptive and innate immune responses are critical for successful anti-viral response (6), and both were described as impaired in severe COVID-19 patients. Namely, reduced functionality and exhaustion of NK cells and CD8^+^ T cells were demonstrated in severe COVID-19 patients (7, 8). On the other hand, CD4^+^T cells specific for either SARS-CoV-2 or other human coronaviruses were found to be critical for successful recovery of the patients and protective immunity against SARS-CoV-2 (8–10). However, due to high variability in T cell responses between different donors (11) it is still not clear how different subsets of T cells correlate with clinical parameters in COVID-19 and its progression. The role of B cells in the pathogenesis of COVID-19 also remains unclear. Although convalescent plasma containing neutralizing antibodies was shown to improve clinical symptoms (12), controversial data on humoral response and immune protection mediated by immunoglobulins have been reported (12, 13).

Impaired adaptive immunity in COVID-19 points to dysregulated innate immune response. Infiltration of neutrophils in the lungs was shown to correlate with poor prognosis in COVID-19 (14). Monocytes producing IL-6 and other inflammatory cytokines are claimed as major inducers of cytokine storm and dysfunctional antigen presentation in this disease (15–17), which provided a basis for an off-label use of tocilizumab in COVID-19 (17). Inflammatory macrophages in the lungs were shown to adopt interferon-signaling and monocyte-recruiting chemokine programs that induce ARDS (15). Therefore, myeloid cells seem to be critical for the regulation of COVID-19, but the exact roles of certain cell subtypes and the underlying immunological mechanisms of dysfunctional immune response remain elusive. Previous findings (18), including our own (19), have shown IL-6 is critical for the induction of myeloid-derived suppressor cells (MDSC) causing immune dysregulation and immune paralysis in cancer. However, the role of MDSCs in progression of COVID-19 and the underlying mechanisms have not been studied thoroughly. A comprehensive immune profiling of COVID-19 patients is still lacking, thus hampering the discovery of common signatures of immune dysfunction in critically ill patients. Thus far, the immunological studies of COVID-19 were mainly focused on either innate or adaptive immune components, so a more complete picture of the immune response in COVID-19, following meta-correlations with the clinical parameters, is largely missing. Therefore, the main aim of this study was to identify the role of key adaptive and innate immune cells, particularly MDSC subsets, involved in COVID-19 pathogenesis and major underlying immunological mechanisms suitable as potential therapeutic targets in COVID-19.

## Materials and Methods

### Patients

Samples of peripheral blood were obtained from 41 COVID-19 patients and 16 healthy volunteers after study approval. Hematological and biochemical analyses of patients and healthy donors were performed at Clinical Hospital Centre (KBC) Zemun and Institute for the Application of Nuclear Energy (INEP), respectively, by using routine clinical laboratory methods. The immunological analyses required safe transportation of blood samples from KBC Zemun to INEP which was performed according to recommendations (20). The study was approved by the Ethical Boards of the KBC Zemun (No. 157/1, from 24.05.2020) and the local Ethical committee at the INEP. All patients and healthy donors provided written informed consents in accordance with the Declaration of Helsinki.

### Flow Cytometry Analysis

For the preparation of white blood cells (WBC) from patients, the aliquots of blood samples were incubated with lysing buffer for 10 min at room temperature to lyse red blood cells, followed by two washes in phosphate buffered saline (PBS) containing 2% FCS and 0.01% Na-azide. Peripheral blood mononuclear cells (PBMCs) were isolated from 6 ml of blood by density centrifugation gradient. For surface labeling, the cells were washed once in PBS containing 2% fetal calf serum (FCS) and 0.01% Na-azide, treated with Human TruStain FcX (Biolegend, San Diego, CA, USA) for 15 min, and then incubated with primary monoclonal antibodies for 30 min at 4°C. Intracellular staining was carried out on PBMCs by using the fixation and permeabilization kit (BD Biosciences) after activation of the cells for 3h with phorbol-12-myristate-13-acetate (20 ng/ml, PMA) and ionomycin (500 ng/ml) and monensin (6 μM) (all from Sigma Aldrich, Darmstadt, Germany). For each analysis, more than 50,000 cells were gated according to their specific side-scatter (SSC)/forward-scatter (FSC), after exclusion of doublets (FScA/FScH) and dead (FSc low) cells. Signal overlap between the channels was compensated before each experiment using single labeled cells, and non-specific fluorescence was determined by using the appropriate isotype control antibodies and fluorescence minus one control. Fluorochrome-conjugated mAbs used in the experiment are shown in **Supplementary Table 1**. The samples were acquired on a BD LSR II flow cytometer (BD Biosciences, San Jose, CA, USA) on the day of sampling and analyzed offline in FCS Express 4 software (De Novo software, Pasadena, CA, USA).

### Mixed Leukocyte Reactions

To assess the suppressive properties of myeloid cells from severe COVID-19 patients, PBMCs from five clinically severe patients were used to isolate monocytic fractions by negative magnetic-activated cell sorting (MACS) with Monocyte Isolation kit (Miltenyi Biotec, Bergisch Gladbach, Germany). Monocytic cells (0.5 × 10^5^–0.124 × 10^5^ cells) were then co-cultivated with allogeneic MACS purified T cells (1 × 10^5^ cells/well) that were isolated from healthy volunteer’s PBMC with Pan T isolation Kit (Miltenyi Biotec). T cells were first labeled with Cell Trace Far Red Cell proliferation kit (Thermo Fisher Scientific, Waltham, MA, USA), according to manufacturer’s protocol. Dynabeads Human T activator CD3/CD28 (Invitrogen, Carlsbad, CA, USA) were added to the co-cultures to stimulate the proliferation of T cells. Control cultures contained only corresponding T cells stimulated with Dynabeads. In some experiments, 5-fluorouracil (5 µM, Ebewe Pharma Ges, Unterach, Austria) was added to monocytic cells/T cell co-cultures. After 5 days of co-cultures, cell culture supernatants were collected for cytokine quantification and the cells were harvested for proliferation assessment. Prior to analysis of proliferation, the samples were stained with 20 μg/ml propidium iodide to exclude dead cells. The Suppression Index was calculated as 1 - (percentage of proliferation in monocytic cells/T cell co-cultures/percentage of proliferation in control T cell culture).

### Quantitative Real-Time PCR

Total RNA was extracted with Trizol reagent (Invitrogen). All samples were treated with DNase I using Ambion DNA-free™ Kit (Thermo Fisher Scientific). Reversed transcription was done with RevertAid RT kit using 200 ng of isolated RNA as a template, according to the manufacturer′s protocol (Thermo Fisher Scientific). Random hexamers (Applied Biosystems, Foster City, CA, USA) and RiboLock RNase inhibitor (Thermo Fisher Scientific) were used in these reactions. Synthesized cDNA was amplified in 7500 real-time PCR system (Applied Biosystems) using IC Green qPCR Universal Kit (NIPPON Genetics, Düren, Germany) under the following conditions: 2 min at 95°C activation, 40 cycles of 5 s at 95°C and 30 s at 60°C. The results were normalized against the *GAPDH* gene and expressed as relative target abundance using the 2^-ΔΔCt^ method. Primers used in the study are presented in **Supplementary Table 2**. All primers were purchased from Thermo Fisher Scientific.

### Western Blot

Simultaneously with RNA extraction, the proteins were isolated from the organic phase of Trizol reagent according to the manufacturer′s protocol (Invitrogen). Protein concentration was measured with BCA protein assay kit (Thermo Fisher Scientific). The extracted proteins (10 μg) were separated on 12% SDS–PAGE and transferred to 0.2 mm nitrocellulose membrane (GE Healthcare, Chicago, IL, USA). Western blotting was performed overnight at 4°C with antibodies against: LC3B (1:2000, Invitrogen, PA1-16930) and GAPDH (1:2,000, Abcam, ab9484, Cambridge, UK). The intensity of the bands was quantified using ImageJ (National Institutes of Health, NIH) software. Samples from nine donors within each clinical group were analyzed for LC3I/LC3II expression by western blot. Additionally, the samples from 15 different donors within each clinical group were analyzed by western blot after puling five samples from different donors into one, thus totally making three puled samples in each clinical cohort.

### Quantification of Cytokines and Immunoglobulins

Cell culture supernatants and/or sera samples collected from COVID-19 patients and healthy volunteers were analyzed using the LEGENDPlex, Human Th Cytokine Panel and Human Inflammatory Panel (both 13‐plex, BioLegend) and the following cytokines were analyzed this way: IL-10, IL-9, IL-6, IL-2, IL-13, IL-5, IL-22, IL-21, IL-4, IL-17F, IL-17A, TNF-α, IFN-γ, IL-1β, IFN-α, MCP-1, IL-8, IL-12p70, IL-18, IL-23, IL-33, according to manufacturer’s instructions. Besides these cytokines, TGF-β, IL-33, and IL-1β concentrations in sera samples were determined by Duo Set ELISA test (R&D Systems, Minneapolis, MN, USA), according to manufacturer’s protocol. The levels of SARS-CoV-2 specific IgG and IgM antibodies were determined by commercial ELISA kits (BioVendor, Brno, Czech Republic). The positivity/negativity of the sera for IgM and IgG was defined according to manufacturer’s recommendations.

### Statistics

Kruskal-Wallis test followed by the Dunn’s multiple comparison test and T-test were used for comparison of clinical, laboratory and immunological parameters between the experimental groups. Values of p<0.05 were considered significant. Principal component analysis (PCA) and Spearman correlation were computed and visualized using RStudio v1.2.5042. PCA was performed on 17 clinical and 144 immune parameters obtained from healthy donors and COVID-19 patients. The data were normalized and used for PCA through the stats package’s prcomp function and plotted using ggbiplot function. To calculate the intersection ellipses in PCA, the overlap function from siar package was used. The potential relationship between 135 parameters collected from total 38 patients were analyzed by calculating of Spearman rank correlation coefficient (rcorr function). In order to analyze the correlation between numerical and categorical variables, the categorical variables (sex, disease severity, mechanical ventilation, outcome) were converted into numerical variables (e.g. female and male sex were assigned with numbers 1 and 2, respectively; disease severity: mild-1 and severe-2; mechanical ventilation: no required-1, required-2; outcome: recovered-1 and exitus letalis-2). Correlation matrix between variables was calculated and visualized as a correlogram using R function corrplot, following hclust for hierarchical clustering order. Calculation of q-values, following false discovery rate (FDR)<0.05 estimation for Spearman correlation test, was performed using qvalue package.

## Results

### Clinical and Laboratory Parameters of COVID-19 Patients

Forty-one patient admitted to KBC Zemun, Belgrade during May and June 2020, who were confirmed COVID-19 cases by qPCR of nasopharyngeal swabs, but without history of autoimmune or malignant illness previously, were included in the study. The clinical parameters for each patient are shown in **Supplementary Table 3**. The most common symptoms were malaise (70.7%), fever (68.2%), dyspnea (41.4%), and cough (39.0%), whereas diarrhea was the rarest symptom (4.8%). Almost all patients had pneumonia (92%) in different stages, either with unilateral (7.8%) or bilateral opacities (92.2%). According to clinical parameters, WHO recommendations (21) and KBC Zemun therapy protocol, twenty-one patients who showed mild to moderate COVID-19 symptoms and required no mechanical ventilation were considered mild. The patients who displayed an increased dyspnea, increased respiration frequency (>30 breath/min), SpO2<93%, PaO2/FlO2<300 and/or at least 50% increased infiltration of lungs within 24–48 h, were considered severe COVID-19 patients. The most common comorbidities were hypertension, cardiovascular diseases, and diabetes type II (**Supplementary Table 3**), and at least one was present in 75% of patients in severe group, in contrast to 53% in the mild group of patients. The patients with mild symptoms received hydroxychloroquine (HCQ) (95.2%) alone or in combination with low molecular-weight heparin (LMWH) (42.8%) and/or antibiotics. These patients developed no complications during hospitalization and were discharged after averagely 13.4 ± 6.4 days in the hospital. In severe group of patients (n=20), treatment also included HCQ (70%), LMWH (100%), and antibiotics. Five patients in this group also received tocilizumab. The course of treatment required intensive ventilation in 45% of severe cases, and the complications such as pneumothorax, pulmonary edema, or *Clostridium difficile* infection, developed in 20% of the severe cases. The total mortality rate in severe group of patents was 40% (8/20), and those recovered from COVID-19 were hospitalized for averagely 20.8 ± 13.7 days, and then either discharged or transferred to another clinic for other illness therapy.

The immunological assessment of COVID-19 patients was performed on average 8.7 day of hospitalization, and the laboratory (hematological and biochemical) parameters performed on that day are shown in **Supplementary Table 4**. Additional experimental group was sex/age matched healthy controls with no history of COVID-19, malignancy, or autoimmune diseases (**Supplementary Figure 1**). Median age for mild COVID-19 patients was 55 years (range 22–78), for severe COVID-19 patients 62.5 years (31–84), and for healthy donors 54 years (26–82). There were no significant differences between the groups in age and gender ratio. Laboratory analyses showed that severe COVID-19 patients had significantly higher number of WBC and neutrophils, compared to healthy donors and patients with mild COVID-19 symptoms. The number of monocytes was above the normal range in 45% of mild and 42% of severe cases, and the average number of monocytes was higher in COVID-19 patients compared to healthy donors. Lymphocytopenia was detected in 38.1% of mild and 80% of severe COVID-19 cases, and the average number of lymphocytes in severe patients’ group was significantly lower when compared to healthy donors and mild patients (**Supplementary Table 4**). Additionally, COVID-19 patients had significantly higher levels of D-dimer, aPTT, fibrinogen, CRP, ferritin and LDH, and in the severe group of patients, these parameters were higher than in mild COVID-19 patients. Moreover, significantly higher levels of AST, ALT, GGT, and urea were observed in severe group patients compared to healthy donors. Also, 84.2% of severe patients displayed hypocalcemia, compared to 27.7% of mild COVID-19 patients.

Cytokine response in severe COVID-19 patients was clearly marked by elevated levels of pro-inflammatory cytokines, including IL-6, IL-8, MCP-1, and IL-18 compared to healthy donors (**Supplementary Figure 2**). However, it was observed that severe COVID-19 patients also display increased levels of TGF-β and IL-10 in their sera, suggesting the activation of immunoregulatory cytokines as well. In contrast, mild COVID-19 patients displayed reduced levels of IL-10 in sera, but the levels of TGF-β were higher than in the sera of healthy donors. The levels of IL-4 in sera of mild COVID-19 patients were slightly lower than in sera of healthy donors. Severe COVID-19 patients displayed reduced levels of IL-12p70, whereas the levels of IL-17A and IL-17F were lower in both mild and severe COVID-19 patients, compared to healthy donors. There were no significant differences in the levels of type-1, type-2 interferons and Th9 polarizing cytokines in the sera of COVID-19 patients (**Supplementary Figure 2**).

### Reduction in T, NK, and NKT Cell Numbers, but not B Cells, Correlate With COVID-19 Severity

**Figure 1:**
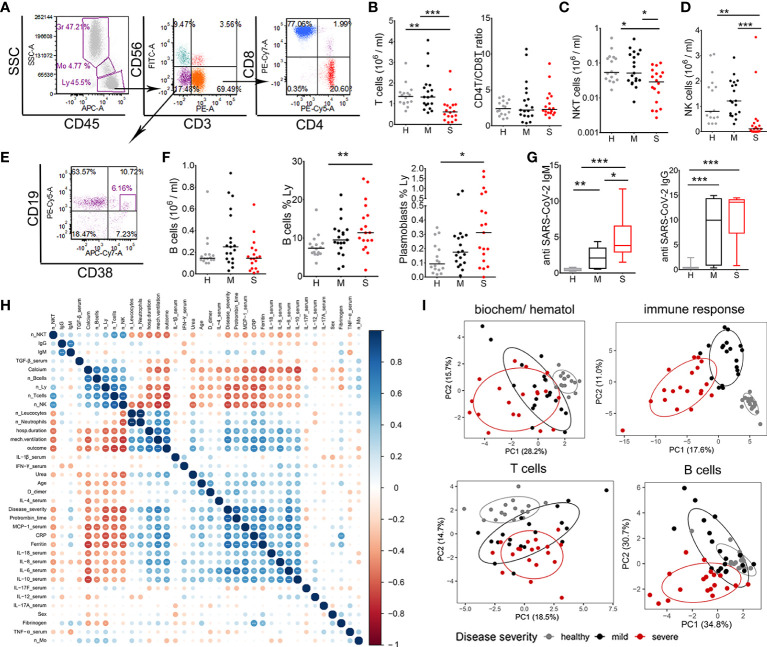
Lymphocytes characterization and correlations with clinical parameters of coronavirus disease (COVID)-19. **(A)** Representative gating of lymphocyte populations is shown. Doublets were excluded by FSc-A/FSc-H (not shown) and lymphocytes (Ly) (CD45^hi^SSc^low^) were gated to identify NKT (CD3^+^CD56^+^), NK (CD3^-^CD56^+^), and T (CD3^+^CD56^-^) cells. T cells were gated to identify CD4 and CD8 T cell subsets. **(B)** The number of T cells/ml was calculated from the percentage of cells and the number of leukocytes for each donor in the groups of healthy donors (H, n=16), mild (M, n=19) and severe (S, n=19) patients. CD4/CD8 T cell ratio was calculated from the percentage of CD4^+^ and CD8^+^ T cells in lymphocytes region. The numbers of NKT **(C)** and NK cells **(D)** were calculated as in **(B)**. **(E)** B cells (CD19^+^) and plasmablasts (CD19^+^CD38^hi^) were gated from CD3^-^CD56^-^ region as indicated. **(F)** The number of B cells/ml, the relative percentage of B cells and plasmablasts in lymphocytes region are shown. **(G)** The levels of SARS-CoV-2-specific IgM and IgG antibodies in sera of patients (mild n=21, severe n=20) and healthy donors (n=16) are shown as Tukey box-whiskers. **(B, D, F, G)** *p < 0.05, **p < 0.01, ***p < 0.005 as indicated (Kruskal-Wallis test with Dunn’s post-test). **(H)** Spearman’s correlation matrix and hierarchical clustering of 36 common features in 19 mild and 19 severe COVID-19 patients. The color and size of the circles represent the correlation coefficient. Asterisks indicate significance levels for each comparison, at FDR below 0.05 (*), 0.01 (**), and 0.001 (***). n_, number; Mo- monocytes. **(I)** Principal component analysis (PCA) of hematological and biochemical (n=17), immune response (n=144), T cells (n=35), B cells and antibody data (n=11) are shown with 95% confidence ellipse for healthy donors, mild, and severe COVID-19 patients.

Lymphocytopenia is the hallmark of COVID-19 correlating with disease severity (5), but it is not clear how the changes in different lymphocytes subsets associate with disease severity. To assess which subpopulation of lymphocytes was mostly affected in COVID-19 patients, flow cytometry analysis was performed from whole blood lysates. The analyses confirmed that the total number of T (CD3^+^CD56^-^) cells was lower in patients with severe COVID-19 symptoms, but not in patients with mild symptoms, compared to healthy donors (**Figure 1A**). The numbers of CD4^+^ T and CD8^+^ T cells were also lower in severe patients (data not shown), but the relative percentage of CD4^+^ T and CD8^+^ T cells and their ratio (**Figure 1B**) was not altered significantly. Besides T cells, lymphocytopenia also affected the total number of NKT (CD3^+^CD56^+^) and NK (CD3^-^CD56^+^) cells in severe patients (**Figures 1C, D**).

Interestingly, we did not observe a reduction in the total number of B (CD3-CD56-CD19^+^) cells, as their relative frequency in lymphocytes’ region increased in patients with severe COVID-19 symptoms. Also, the relative frequency of antibody-producing CD19^+^CD38^hi^ plasmablasts (PB) was significantly higher in the group of severe COVID-19 patients. The serological analysis confirmed the presence of specific antibodies against SARS-CoV-2 in all patients with severe symptoms and 86% of mild cases. In severe patients’ group, 95% (19/20) had IgM and 100% IgG type of antibodies in the sera. In the group of mild patients, 67% (14/21) had IgM class antibodies and 76.2% (16/21) had IgG antibodies specific for SARS-CoV-2. Measured levels of IgM class antibodies in severe patients’ group were significantly higher than in the mild group of patients, whereas the levels of IgG were somewhat higher in severe group of patients but not significantly. There were no detectable IgM antibodies in the group of healthy donors, one donor had detectable IgG (**Figure 1G**).

To assess the potential association between immunological parameters and clinical features of COVID-19 patients we preformed consensus hierarchical clustering of Spearman correlation analyses between most common COVID-19 parameters (36 variables, **Figure 1H**), and between key parameters reported in this study (135 variables, **Supplementary Figure 3**). The analysis showed strong positive correlations between clinical parameters (disease severity, duration of hospitalization, mechanical ventilation, outcome of the disease), biochemical parameters (prothrombin time, CRP, ferritin, calcium), hematological (number of neutrophils and WBC) and immunological parameters (MCP-1, IL-6 and IL-10). As shown in the red clusters of correlogram, the total number of T, NK, NKT cells in patients’ blood strongly negatively correlated with the clinical/biochemical/immune parameters pointing to disease progression. In contrast, number of B cells and the levels of IgM and IgG antibodies correlated poorly with other parameters analyzed (**Figure 1H**, **Supplementary Figure 3**). Overall, the parameters of immune response analyzed (n=144) clearly separated patients into clinical cohorts much better than standard biochemical/hematological parameters (n=17) (**Figure 1H**), suggesting that the parameters of immune response have a stronger prediction value for COVID-19 severity. PCA based on T cell parameters (n=35) showed clustering of healthy, mild, and severe COVID-19 patients in PCA space with partial overlap between mild and severe patients. The overlap in PCA space between healthy and COVID-19 patients was smaller for T cell parameters than the overlap for B cell parameters and humoral response (n=11). These results suggested that lymphocytopenia in COVID-19 severely affects the number of NK, NKT and T cells, but not B cells. Moreover, the impairment of T cell number and their functions seems more clinically relevant for COVID-19, than the changes in B cell response.

### T Cell Activation, Induction Of Type 1 Response, and Effector Memory CD4^+^T Cell Response Are Impaired in Severe COVID-19 Patients

**Figure 2:**
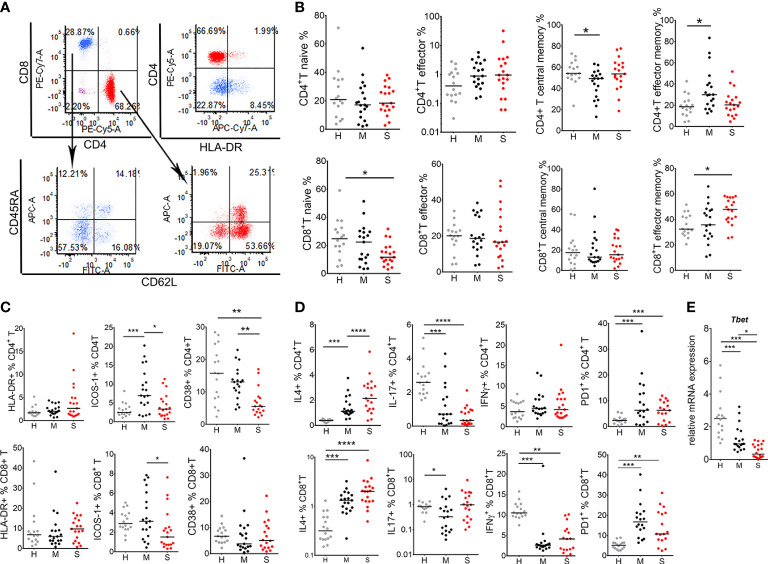
T cell response in coronavirus disease (COVID)-19 patients and healthy donors. **(A)** Gating strategy for identification of T cell subsets is shown. After exclusion of doublets, T cells were gated from lymphocytes (CD45^hi^SSc^low^) region as CD3^+^CD56^-^ cells, followed by CD4 and CD8 phenotyping. **(B)** The percentages of naïve (CD62L^+^CD45^+^), effector (CD62L^-^CD45RA^+^), central memory (CD62L^+^CD45RA^-^) and effector memory (CD62L^-^CD45RA^-^) T cells, gated from CD4^+^ T cell (red) or CD8^+^ T cell (blue) region as in **(A)**, are shown as the proportion of total CD4^+^ or CD8^+^ T cells. **(C)** The percentages of HLA-DR^+^, ICOS-1^+^, and CD38^+^, and **(D)** IL-4^+^, IL-17^+^, IFN-γ^+^ and PD1^+^ CD4^+^ T, and CD8^+^ T cells are shown as the proportions of total CD4^+^T or CD8^+^T cells as indicated. **(E)** Relative mRNA expression of *T-bet* in PBMC is shown. **(B–E)** The results for each donor in the groups of healthy donors (H, n=16), mild (M, n=19) and severe (S, n=19) patients, and the corresponding medians are shown. *p < 0.05, **p < 0.01, ***p < 0.005, ****p < 0.001 as indicated (Kruskal-Wallis test with Dunn’s post-test).

Considering previous results, next we analyzed subtypes and functions of T cells in COVID-19 patients and healthy donors in more details (**Figure 2**). The analysis of CD4^+^ and CD8^+^ T cells according to CD45RA and CD62L expression identified naïve (CD62L^+^CD45RA^+^), effector (CD62L^-^CD45RA^+^), central memory (CD62L^+^CD45RA^-^) and effector memory (CD62L^-^CD45RA^-^) T cells. These T cell subsets were analyzed both as the relative percentage of total CD4 or CD8 T cells (**Figures 2A, B**), or as the total number of these cells (**Supplementary Figure 4**). Lymphocytopenia in the group of severe patients significantly affected central memory CD4 ^+^ T cells, and all subsets of CD8^+^ T cells, especially naïve CD8^+^ T cells. In CD8^+^ T cell compartment, severe COVID-19 patients displayed lower percentage of naïve CD8 cells but an increased percentage of effector memory CD8^+^ T cells, compared to healthy donors. The percentages of effector CD8^+^ T cells and central memory CD8^+^ T cells were not different significantly between the groups. In CD4^+^ T cell compartment, a lower percentage of central memory T cells was observed along with an increase in both percentage and the number of effector memory CD4^+^ T cells in the mild group of patients, compared to healthy donors. No increase in effector memory CD4^+^ T cells was observed in the group of severe COVID-19 patients.

As a markers of T cell activation, we analyzed HLA-DR (22), ICOS-1 (23) and CD38 (11) expression on T cells. There were no significant differences in the percentage of HLA-DR^+^ T cells between the groups. In contrast, mild COVID-19 patients displayed increased ICOS-1 expression on CD4^+^T cells (**Figure 2C**). However, lower percentages of ICOS-1^+^CD8^+^ T, ICOS-1^+^CD4^+^ T, and CD38^+^CD4^+^ T cells were observed in severe COVID-19 patients (**Figure 2C**). Intracellular staining of T cells for cytokines showed that the percentage of IFN-γ-producing CD8^+^ T (Tc1) cells was reduced, whereas the percentage of IL-4-producing CD8^+^ T (Tc2) increased in both mild and severe COVID-19 patients compared to healthy donors, suggesting an impaired type 1 response in CD8 T cells. The frequency of Th17 cells (CD4^+^IL-17^+^) was reduced in both mild and severe COVID-19 group, whereas the frequency of Th2 cells (CD4^+^ IL-4^+^) was higher in both COVID-19 groups, compared to healthy donors. The percentage of Th1 (CD4^+^IFN-γ^+^) cells was not altered in COVID-19 patients, although the total number of Th1 cells was lower in severe COVID-19 patients compared to healthy donors due to lymphocytopenia (data not shown). CD4 and CD8 T cells had increased expression of PD1 in both groups of COVID-19 patients. Quantitative PCR analysis performed on total PBMCs suggested the total reduction of *T-bet* mRNA levels in COVID-19 patients compared to control, especially in severe COVID-19 group (**Figure 2D**). These results indicate that the activation of T cells, induction of type 1 immune response, and the response of effector memory CD4+ T cells, is impaired in COVID-19 patients, especially in those with severe clinical manifestations.

### Antigen Presentation and Autophagy Are Significantly Impaired in COVID-19 Patients

**Figure 3:**
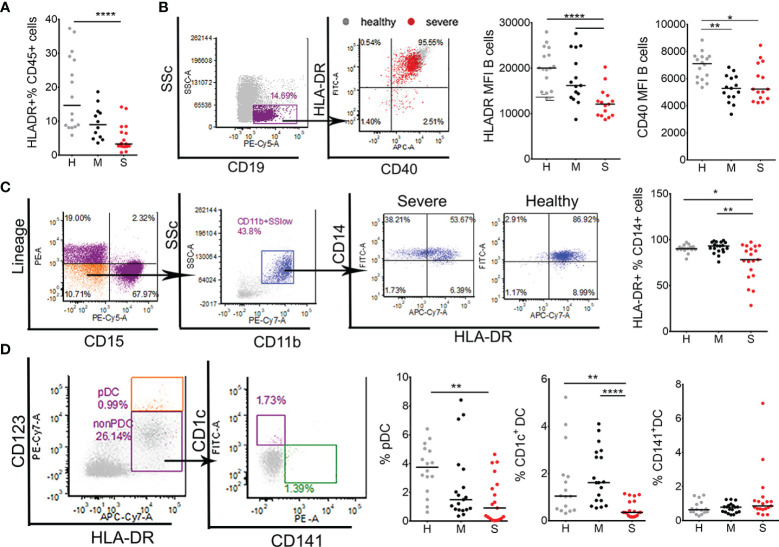
Characterization of antigen presenting cells in coronavirus disease (COVID)-19 patients and healthy donors. **(A)** The percentages of total HLA-DR^+^ cells within CD45^+^ leukocytes were obtained by flow cytometry in healthy donors (H, n=16), mild (M, n=12), and severe (S, n=19) COVID-19 patients. **(B)** The expressions of HLA-DR and CD40 were analyzed on B cells gated from PBMC as SSc^low^CD19^+^ cells. CD40/HLA-DR dot-plot represent an overlay of flow cytometry data from one healthy donor (gray) and one severe patient (red), and the summarized results from 16 healthy donors,15 mild, and 15 severe patients are shown as mean fluorescence intensity (MFI). **(C)** HLA-DR expression on total monocytes was analyzed after gating these cells from CD45^+^WBC as linage (lin)^-^ (CD3^-^CD7^-^CD19^-^CD56^-^CD123^-^Glycophorin A^-^) CD15^-^ CD11b^+^ CD14^+^ cells. Representative CD14/HLA-DR dot-plots are shown from one severe patient and one healthy donor, and the summarized results on the percentage of HLA-DR^+^ cells of total CD14^+^ cells is shown. **(D)** Blood DC subsets were analyzed from monocytes region of PBMC as the percentage of pDC (HLA-DR^+^CD123^hi^), CD1c^+^DC (HLA-DR^+^CD123^low/-^CD1c^+^CD141^-^), and CD141^+^DC (HLA-DR^+^CD123^low/-^CD1c^-^CD141^+^). The summarized results are expressed as the percentage of gated cells within monocytes region. **(C, D)** Data from 16 healthy (H) donors, 19 mild (M), and 19 (S) severe COVID-19 patients are shown. *p < 0.05, **p < 0.01, ****p < 0.001 (Kruskal-Wallis test with Dunn’s post-test).

Impaired T cell response suggests that antigen presentation and the activation of antigen presenting cells (APC) might be impaired. Indeed, the total expression of HLA-DR^+^ cells was reduced in severe COVID-19 patients compared to healthy donors (**Figure 3**). Additionally, HLA-DR expression was analyzed on key APC populations including B cells, DC and monocytes. HLA-DR and CD40 expression were significantly lower in B cells of severe COVID-19 patients compared to B cells in healthy donors (**Figure 3B**). Mild COVID-19 patients displayed a reduced expression of CD40, but not HLA-DR on B cells. Monocytes also displayed significantly reduced expression of HLA-DR in severe COVID-19 patients (**Figure 3C**). Blood DC were identified as HLA-DR^+^CD123^hi^ plasmacytoid (p)DC, CD1c^+^mDC (HLA-DR^+^CD123^low^CD1c^+^CD141^-^, DC2) and CD141^+^mDC (HLA-DR^+^CD123^low^CD1c^-^CD141^+^, DC1) (**Figure 3D**). Severe COVID-19 patients contained lower proportions of CD1c^+^ mDC compared to mild patients, and lower proportions of pDC and CD1c^+^ mDC compared to healthy donors. In contrast, the proportion of CD141^+^ mDC was not significantly different between the groups. In addition, we found that the mRNA expression of key signal transducing and transcription factors involved in activation of APC and other immune cells, such as MyD88, NF-kB, RANKL and STAT-1, were significantly reduced in PBMC of severe COVID-19 patients, compared to healthy donors and mild COVID-19 patients. In contrast, mRNA expression of osteopontin (OPN) was highly up-regulated in severe COVID-19 patients (**Supplementary Figure 5**).

**Figure 4:**
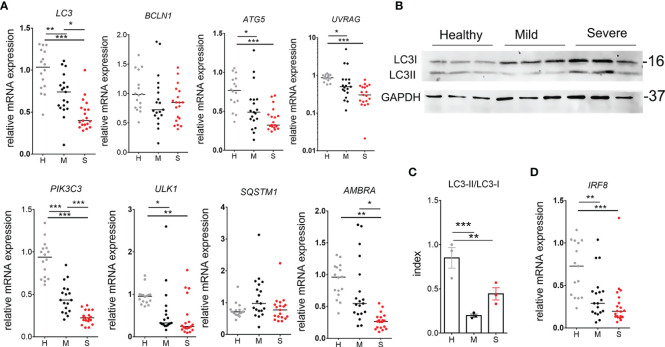
Autophagy analysis in peripheral blood mononuclear cells (PBMC) of coronavirus disease (COVID)-19 patients and healthy donors. **(A)** Relative mRNA expressions of LC3, BCLN1, ATG5, UVRAG, PIK3C3, UL1, SQSTM1, and AMBRA were analyzed in freshly isolated PBMC of 16 healthy donors (H), 19 mild (M) and 20 severe (S) COVID-19 patients by qPCR. **(B)** Western blot of LC3I (16kDa), LC3II, and GAPHD (37kDa) is shown, wherein each blot in a column represent a sample prepared by puling equivalent amounts of proteins isolated from PBMC of 5 different donors (totally 15 donors per group). **(C)** The Index of LC3II/LC3I conversion shown for these samples, was calculated by densitometry. *p < 0.05, **p < 0.01 as indicated (Student t-test). **(D)** Relative mRNA expression of IRF-8 was analyzed as described in **(A)**. **(A, D)** *p < 0.05, **p < 0.01, ***p < 0.005 as indicated (Kruskal-Wallis test with Dunn’s post-test).

The presentation of antigens restricted by MHC II is tightly regulated by autophagy (24). Moreover, autophagy is critically involved in the activity of myeloid cells promoting tolerance and anti-inflammatory processes (24–26). Therefore, we evaluated the level of autophagy related genes in PBMC of COVID-19 patients and healthy donors (**Figure 4A**). It was found that transcript levels of key factors promoting autophagy, i.e. *ULK-1*, *ATG5*, *UVRAG*, *AMBRA, PIK3C3*, and *LC3*, are significantly reduced in mononuclear cells of COVID-19 patients compared to healthy controls, and the phenomenon was more pronounced in severe COVID-19 patients. Thereby, the relative expression of *BECN1* (Beclin-1) and *SQSTM1* (p62) mRNAs were not altered significantly, but they positively correlated with the expression of other autophagy markers (**Supplementary Figure 3**). Additionally, the LC3II/LC3I protein expression ratio as an indicator of autophagy, was evaluated in 9 patients within each group using western blot analysis (**Supplementary Figure 6**). Alternatively, to achieve higher sensitivity in protein expression, 3 samples were generated for each cohort group, each sample containing equivalent amount of proteins from 5 different donors (15 donors in total) within that group (**Figure 4B**). The index of LC3II/LC3I was significantly reduced in both mild and severe COVID-19 patients compared to healthy donors, suggesting that the autophagy was reduced in COVID-19 patients (**Figure 4C**). Furthermore, interferon regulatory factor (IRF)8 seems to be critically involved in stress induced regulation of autophagy, antigen presentation and efficient clearance of intracellular pathogens, among other functions (27). In line with this, we found that mRNA levels of *IRF8* in mild and severe COVID-19 patients were significantly reduced compared to healthy donors (**Figure 4D**). These results suggested that reduced proportions of APCs in PBMC and generally reduced HLA-DR expression in COVID-19 patients could be related to reduced expression of IRF-8, autophagy-related genes and altered myeloid cell differentiation.

### Monocytic MDSCs in Severe COVID-19 Patients Contribute Mostly to IL-6 Production

**Figure 5:**
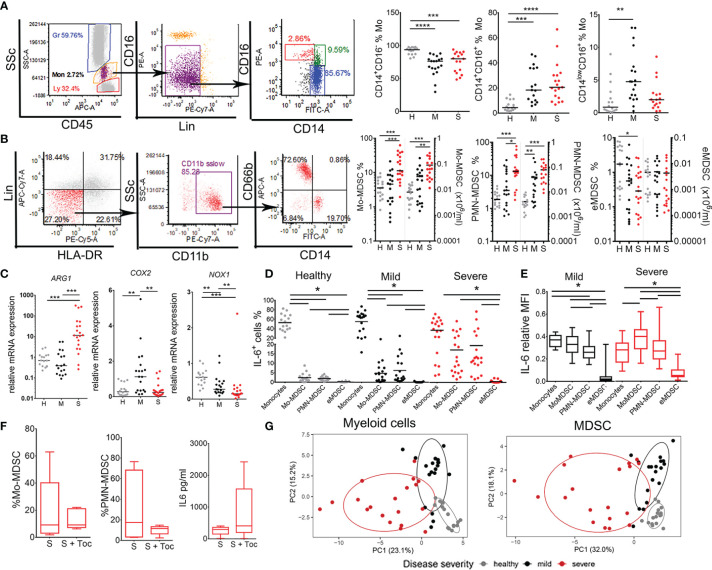
Analysis of monocytes and myeloid derived suppressor cells (MDSC) subsets in coronavirus disease (COVID)-19 patients and healthy donors. **(A)** Classical (CD14^+^CD16^-^), inflammatory (CD14^+^CD16^+^), and transitory (CD14^low/-^CD16^+^) monocytes (Mo) were identified in monocytes region (CD45^mid^SSc^mid^) after excluding doublets, CD16^hi^ granulocytes and lineage (lin)^+^ (CD3^+^CD7^+^CD19^+^CD56^+^CD123^+^Glycophorin A^+^) cells. The summarized results are expressed as the proportion of cells in gated monocytes. **(B)** MDSC were gated from monocytes region of PBMC as Lin^-^ (CD3^-^CD7^-^CD19^-^CD56^-^CD123^-^Glycophorin A^-^) HLA-DR^low/-^ CD11b^+^ cells expressing either CD66b/CD15 (PMN-MDSC), CD14 (Mo-MDSC) or neither CD66b/CD15 nor CD14 marker (eMDSC). The summarized results are expressed as the proportion of cells in monocytes region and the total number of cell/ml was calculated based on the hematological count of monocytes. **(C)** Relative mRNA expressions of ARG-1, COX2, and NOX1 were analyzed in PBMC. **(D)** The percentages of IL-6^+^ monocytes, Mo-MDSC, PMN-MDSC, and eMDSC (gated as in **A, B**) are shown as the proportion of cells in monocytes region. **(A–D)** Summarized data were shown for 16 healthy donors (H), 19 mild (M), and 19 severe (S) patients. *p < 0.05, **p < 0.01, ***p < 0.005, ****p < 0.001, as indicated (Kruskal-Wallis test with Dunn’s post-test). **(E)** Relative expression of IL-6 in Monocytes and MDSC subsets of 19 mild and 19 severe patients was calculated by dividing mean fluorescence intensity (MFI) of IL-6 in each cell subset and the sum of IL-6 MFIs in all subsets. The results are shown as Tukey box-whiskers. **(D, E)** Horizontal lines between the histograms indicate statistical significance at p<0.05 (Kruskal-Wallis test with Dunn’s post-test). **(F)** The percentages of Mo-MDSC, PMN-MDSC and sera levels of IL-6 from 5 severe COVID-19 patients receiving Tocilizumab as a therapy (S + Toc) and age/gender matched severe patients who did not (S) are shown as Tukey box-whiskers. **(G)** Principal component analysis (PCA) of myeloid cells parameters (n=62) and MDSC parameters (n=27) is shown with 95% confidence ellipse for healthy donors (gray), mild (black) and severe (red) COVID-19 patients.

IL-6 was shown to down-regulate MHC II expression and IL-12 production by DC (28), which might explain the findings on impaired induction of type 1 response in T cells and diminished HLA-DR expression in COVID-19. The most important sources of IL-6 reported in COVID-19 were myeloid cells (17, 29), but it remained unclear which subtype of myeloid cells predominantly contributes to IL-6 production. Therefore, we analyzed changes in myeloid cell populations and their IL-6 expression. The analyses of monocytes subpopulations including classical (CD14^+^CD16^-^), inflammatory (CD14^+^CD16^+^) and transitory (CD14^low^CD16^+^) monocytes suggested that the percentage of inflammatory monocytes was significantly increased in COVID-19 patients compared to healthy donors (**Figure 5A**). This increase correlated with a lower relative frequency of classical monocytes (CD14^+^CD16-). Interestingly, the patients with mild COVID-19 symptoms, unlike severe COVID-19 patients, displayed an increased frequency of transitory monocytes (CD14^low^CD16^+^) compared to healthy donors.

MDSC populations were analyzed after the isolation of PBMC by density gradient. According to recommendations (30), MDSC were identified as Lin^-^HLA-DR^low^ CD11b^+^ SSc^low^ cells that express either CD15 or CD66b^+^ (PMN-MDSC), CD14^+^ (Mo-MDSC), or neither CD14 nor CD15/CD66b (early (e)MDSC) (**Figure 5B**). An increased frequency and the total number of both Mo-MDSC and PMN-MDSC was detected in severe group of COVID-19 patients compared to both healthy donors and mild patients. The proportion of eMDSC was low in the examined samples. A significant positive correlation between the proportion of Mo-MDSC and PMN-MDSC in COVID-19 patients was observed (**Supplementary Figure 3**), suggesting that the proportion of these cells increased in parallel in COVID-19. These results correlated with increased total expression of Arginase 1 (*ARG-1)* mRNA in PBMC of severe COVID-19 patients (**Figure 5C**). The expression of Prostaglandin-endoperoxide synthase 2 (*PTGS2*, also known as cyclooxygenase 2) mRNA was increased in PBMC of mild, but not severe COVID-19 patients, whereas the expression of NADPH oxidase (*NOX-1*) mRNA was reduced in both mild and severe COVID-19 patients compared to healthy donors.

Next, we evaluated which myeloid cell type mostly express IL-6. The percentage of IL-6^+^ cells in healthy donors was the highest in the population of monocytes (**Figure 5D**). However, in COVID-19 patients, significantly higher contribution of IL-6-producing cells came from Mo-MDSC and PMN-MDSC (**Figure 5D**). Thereby, in severe group of patients the relative contribution of IL-6-producing Mo-MDSC and PMN-MDSC was no longer different from that of IL-6-producing monocytes. Moreover, the relative expression of IL-6 in severe COVID-19 patients was the highest in Mo-MDSCs, as compared to the relative expression of IL-6 in monocytes and PMN-MDSC (**Figure 5E**). The sera IL-6 levels in COVID-19 patients correlated positively with the percentage of Mo-MDSC and somewhat lesser with PMN-MDSC, but negatively with the percentage of monocytes (**Supplementary Figure 3**), suggesting that Mo-MDSC mostly contribute to serum IL-6 in severe COVID-19 patients.

Considering that MDSC expansion and non-specific chronic inflammation are mediated by IL-6 (17–19), we analyzed the effects of Tocilizumab in COVID-19 patients, 5 of which who received Tocilizumab prior to immunological assessment and 5 age/sex/severity of disease-matched patients who did not. The results suggested that Tocilizumab therapy did not affect the percentage of Mo-MDSC, PMN-MDSC, nor the sera IL-6 levels in severe COVID-19 patient group (**Figure 5F**). PCA performed on all myeloid cells (n=62 parameters) and MDSC alone (n=27 parameters), showed clear clustering of severe, mild, and non-COVID-19 patients with minimal overlap in PCA space (**Figure 4G**). These results suggest that myeloid cells, especially MDSC, critically determine the severity of COVID-19, much more than T and B cells (**Figure 1**).

### Monocytic MDSC and Regulatory T and B Cell Subsets Accumulate in Severe COVID-19 Patients

**Figure 6:**
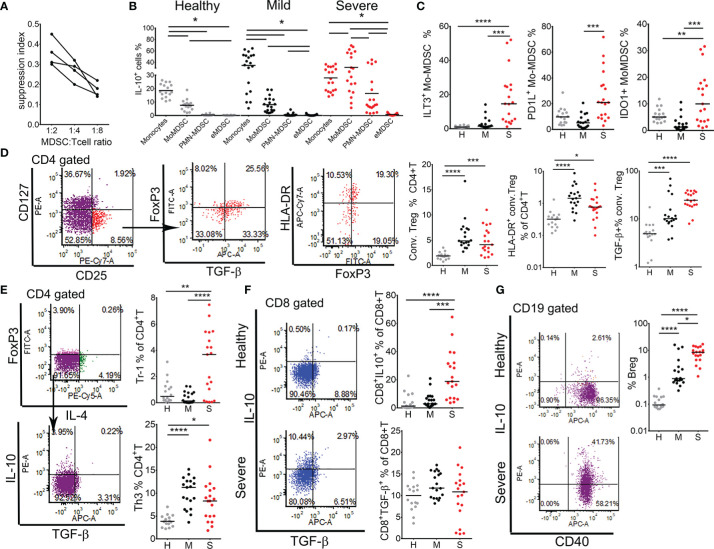
Analysis of regulatory mechanisms in myeloid derived suppressor cells (MDSC) and regulatory lymphocytes. **(A)** Suppression capacity of monocytic cells (0.5 × 10^5^, 0.25 × 10^5^, 0.12 × 10^5^ cells/well) isolated from four severe patients by MACS was tested in co-culture with allogeneic MACS purified T cells (1 × 10^5^) from a healthy donor treated with CD3/CD28 beads. The suppression index was calculated as 1 - % of T cells proliferation in co-culture with monocytic cells, divided by % of T cells proliferation in control cultures without monocytic cells. **(B)** The percentages of IL-10^+^ monocytes, Mo-MDSC, PMN-MDSC and eMDSC (gated as in A and B in **Figure 5**) in healthy donors, mild, and severe COVID-19 patients are shown. Horizontal lines between the histograms indicate statistical significance at p<0.05. **(C)** The percentages of ILT-3^+^, PD-L1^+^, and IDO-1^+^ Mo-MDSC, gated as in **Figure 5B**, are expressed as the proportion of cells in monocytes region. **(D)** Conventional (conv.) Tregs were gated from CD4^+^T cells as CD127^-^CD25^+^FoxP3^+^ cells. Total percentages of conv. Tregs and HLA-DR^+^ (activated) Tregs are shown as the proportion of CD4^+^T cells. The percentage of TGF-β+ cells is shown as the proportion of total conv. Tregs. **(E)** Type 1 regulatory (Tr-1) and Th3 cells were gated as CD4^+^FoxP3^-^IL4^-^IL-10^+^ and CD4^+^FoxP3^-^IL4^-^TGF-β^+^ cells, respectively. **(F)** Expression of IL-10 and TGF-β were analyzed in CD8^+^ T cells and representative dot-plots from one healthy and one severe patient are shown. **(G)** B regulatory (Breg) cells were gated as CD19^+^CD40^+^IL-10^+^ cells and representative dot-plots from a healthy and a severe patient are shown. **(C–G)** Summarized data from 16 healthy donors (H, gray dots), 19 mild (M, black dots), and 19 severe (S, red dots) COVID-19 patients are shown as the percentage of gated lymphocytes or as indicated. *p < 0.05, **p < 0.01, ***p < 0.005, ****p < 0.001 as indicated (Kruskal-Wallis test with Dunn’s post-test).

MDSC, especially Mo-MDSC, display abundant immune suppressive mechanisms (30, 31). Monocytic cells isolated from PBMC of four severe COVID-19 patients exhibited suppressive activity on the proliferation of T cells stimulated with CD3/CD28 beads (**Figure 6A**). Therefore, we next analyzed which regulatory mechanisms are displayed by monocytes and MDSC. It was found that in mild COVID-19 patients, monocytes significantly increased IL-10 expression compared to healthy donors, and they were the dominant producers of IL-10 (**Figure 6B**). In contrast, Mo-MDSC were the dominant producers of IL-10 in severe COVID-19 patients. PMN-MDSC also increased IL-10 production in severe patients, but the total frequency of IL-10^+^ PMN-MDSC was much lower than IL-10^+^ Mo-MDSC (**Figure 6B**). Moreover, the total percentages of ILT3^+^, PD-L1^+^ and IDO-1^+^ Mo-MDSC were increased in severe COVID-19 patients compared to both mild patients and healthy donors (**Figure 6C**). The expression of PD-L1, ILT-3 and IDO-1 in monocytes, as well as their total number, was reduced in severe patients, and their expression in PMN-MDSC and eMDSC was low (data not shown).

An increased expression of PD-L1 in the expanded Mo-MDSC could contribute to the observed reduction of CD8^+^ T cell activation and their exhaustion (**Figure 2**). On the other hand, we showed previously that IL-10, ILT-3 and IDO-1 are involved in the induction of different subtypes of regulatory T cells (19, 25, 32). Therefore, we next analyzed PBMC isolated from healthy donors and patients groups for the presence of activated conventional Tregs (CD4^+^CD127^-^CD25^hi^HLA-DR^+^), suppressor CD8^+^ Tregs (CD8^+^IL-10^+^), Tr-1 (CD4^+^FoxP3^-^IL-4^-^IL-10^+^), Th3 (CD4^+^FoxP3^-^IL4^-^TGF-β^+^) cells, and Bregs (CD19^+^IgM^+^IL10^+^). The percentage of conventional Tregs was higher in both mild and severe COVID-19 patients compared to healthy donors. Additionally, the percentage of TGF-β-producing conventional Tregs was the highest in severe patients. IL-10-producing Tr1 cells (**Figure 6E**) and suppressor CD8^+^T cells (**Figure 6F**) were also increased in severe COVID-19 patients, whereas TGF-β-producing Th3, but not CD8^+^ T cells (**Figure 6E**), were increased in both mild and severe patients. The proportion of IL-10-producing Bregs increased significantly in mild COVID-19 patients, and more so in severe COVID-19 patients compared to healthy donors (**Figure 6G**). These results suggested that MDSC and regulatory lymphocytes could be critically involved in immune suppression in severe COVID-19 patients mediated by IL-10- and TGF-β-dependent mechanisms. Moreover, hierarchical clustering analysis showed that the expansion of MDSC populations positively correlated with clinical, biochemical, and immunological markers of COVID-19 severity, particularly B and T regulatory subsets, and negatively correlated with markers of antigen presentation, autophagy, lymphocytes number and induction of type 1 immune response (**Supplementary Figure 3**).

### Depletion of MDSC Might Provide Clinical Benefits for Severe COVID-19 Patients

The results indicated that MDSC could be critical pathogenic drivers of inflammation and immune suppression in COVID-19, and the main targets for potential therapy. Previous studies showed that 5-fluorouracil (5-FU) applied in small doses, selectively kill MDSC and enhances Th1 cell response (33). Therefore, in a pilot study we evaluated the effects of 5-FU *in vitro* by treating co-cultures of MACS sorted monocytic cells of 4 severe COVID-19 patients and purified allogeneic T cells in the presence of CD3/CD28 immunobeads. The monocytic cells contained increased percentage of either Mo-MDSC, PMN-MDSC or both. In line with previous findings (33), the percentage of apoptotic monocytic cells increased in co-cultures treated with 5-FU after 3 days of cultivation, as compared to control non-treated co-cultures (data not shown). The supernatants collected from the co-cultures showed significantly decreased levels of IL-8, Th2 cytokines (IL-13 and IL-5), Th17 cytokines (IL-22, IL17F and IL17A), as well as IL-10 after the treatment with 5-FU (**Supplementary Figure 7**). Moreover, we observed increased levels of IL-2 and IFN-γ in 5-FU treated co-cultures compared to control co-cultures. These results suggested that 5-FU could potentially recover lymphocytes proliferation and propagate Th1-mediated immune response by lowering the burden of MDSC.

## Discussion

Dysregulated immune response is the hallmark of severe COVID-19 (7, 11, 17). However, it remained unclear which adaptive and innate immune cells are critically involved in COVID-19 pathogenesis, and what immunological mechanisms could be useful targets for therapy. To this aim, we have analyzed ~140 immunological parameters of cellular and humoral immune response in 41 COVID-19 patients divided into clinically mild and severe cohort group, along with 16 healthy donors. Present comorbidities were heterogenous within both mild and severe patients, so they could not account for all the immunological differences observed in these patients. Patients with history of malignancy or autoimmune disease were not included in the study to avoid non-COVID-19-induced alterations of the immune response. Additionally, the protocol for COVID-19 patients treatment used (**Supplementary Table 3**) did not include corticosteroids, which are known as strong modulators of the immune system (34).

Besides lymphocytopenia and neutrophilia which are known indicators of severe COVID-19 (17), we found that increased WBC count can also indicate for poor disease outcome, increased need for mechanical ventilation and longer duration of hospitalization. These results contrast other reports showing no significant changes in WBC count in COVID-19 patients (5, 11). Biochemical analyses confirmed that markers of inflammation, including CRP, Ferritin, LDH, and D-dimer have good prognostic value for disease progression (35). In addition, we found that calcium levels are reduced in severe COVID-19 patients, and this parameter showed strong negative correlations with MCP-1, IL-18, IL-8, IL-6, and IL-10 and positive correlation with T cell number, indicating its good prognostic factor. Although hypocalcemia was previously shown as an independent risk factor for COVID-19 (36), we showed for the first time strong correlation between calcium levels in sera and efficient immune response in COVID-19. Hypocalcemia could be associated with vitamin D deficiency, another risk factor for COVID-19 (37). Therefore, calcium and vitamin D supplementation could potentially improve the therapy of COVID-19 patients. Taken together, biochemical and hematological data combined clearly separated healthy donors from COVID-19 patients in PCA space, but PCA based on immunological parameters analyzed in this study segregated patients much better for the severity of the disease, pointing to critical role of the immune system in COVID-19 pathogenesis.

Lymphocytopenia affected different subpopulations of lymphocytes, including CD4^+^ and CD8^+^ T cells, NK and NKT cells, but not B cells subsets, as their relative percentage within lymphocytes increased in severe COVID-19 patients. Giamarellos-Bourboulis et al. (17) found unchanged counts of CD45^+^CD19^+^ lymphocytes in 14 COVID-19 patients with dysregulated immune phenotype compared to 10 healthy controls, which is in line with our observations. However, they also found lower count of B cells in three patients with macrophage activated syndrome and nine patients without immune dysregulation phenotype, suggesting that different stages of COVID-19 can significantly alter the number of B cells in blood. Mathew et al. (11) found that 2/3 of COVID-19 patients displayed pronounced B cell response characterized by a relative increase in IgD^-^CD27^-^ B cells and CD27^+^CD38^+^ plasmablasts, which correlated with lower T cell count and increase in inflammation markers, and similar finding were reported in other studies (38). Relative increase in the number of plasmablasts also characterize dysregulated immune response in Denga virus (39) and Ebola infections (40). In contrast to these studies, our results suggested that the increase in relative percentage of B cells and plasmablasts do not correlate with poor disease outcome or any other clinical parameter (**Supplementary Figure 3**), so this phenomenon could rather be a consequence of reduced frequency of other lymphocyte subsets in COVID-19. A higher prevalence of IgM and IgG antibodies in severe patients could be explained by a longer period of their hospitalization before blood sampling (**Supplementary Table 3**). However, it is not clear why the increase in IgM and IgG antibodies specific for SARS-CoV-2 were not protective in COVID-19 patients. A possible explanation is that SARS-CoV-2 preferentially utilizes cell-to-cell infections, rather than extracellular pathway for its dissemination, making the antibody response less effective. *In vitro* studies showing that SARS-CoV-2, much more than SARS, utilizes cell-to-cell transport (41), as well as dissemination *via* peripheral nerves to induce multi-organ infection (42), support this hypothesis. On the other hand, the clinical benefits of convalescence plasma therapy could be ascribed to neutralization of cytokines, complement and autoantibodies, in addition to the presence of neutralizing SARS-CoV-2 antibodies (43). The levels of anti-SARS-CoV-2 antibodies in plasma also did not correlate with poor disease outcome, suggesting that IgM, IgG antibodies specific for SARS-CoV-2 N antigen and B cells are not drivers of COVID-19 pathogenesis. We did not examine the levels of S domain RBD-specific neutralizing antibodies, which were described as important in COVID-19 (44). Therefore, additional investigations of humoral immune response, especially in longitudinal studies, are necessary to better delineate the role of antibodies in this disease.

Previous reports suggested that antigen-specific T cells were identified in central memory or effector memory populations (10, 45). Our results showed increased percentage of T cells in COVID-19 patients exclusively in effector memory (CD62L^-^CD45RA^-^) subsets within both CD4^+^ and CD8^+^ T cell compartments. However, it seems that the effector memory T cells involved in the response in COVID-19 patients came from different precursors and that this is related to disease severity. Namely, in mild patients an increase in effector memory CD4^+^T cells was accompanied by a relative reduction in central memory CD4^+^T subsets. These results suggest that a subset of memory CD4^+^ T cells could have reacted to novel SARS-CoV-2 effectively, enabling a more efficient immune response in these patients. In line with this, it has been shown that 20-50% of people who never encountered SARS-CoV-2 contained specific memory CD4^+^ T cells, but not memory CD8^+^T cells, which could react to SARS-CoV-2 due to their cross-reactivity with other human coronaviruses (45). This phenomenon could also explain the presence of IgG antibodies in sera of a healthy donor in our study, but to prove these hypotheses the measurements of antigen-specific T cell subsets are necessary in future studies. In contrast to CD4^+^ T cells, we found a reduction of naïve CD8^+^T cells that was followed by a relative increase in percentage, but not in total number of effector memory CD8^+^T cells, suggesting that naïve CD8^+^T cell could have been the dominant responders in severe COVID-19 patients. Considering a substantial reduction in the repertoire of naïve T cells during aging (46), naïve T cell response could be ineffective in elderly COVID-19 patients. Lymphocytopenia in severe COVID-19 patients reduced the total number of effectors CD8^+^ T cell subsets and the analysis of T cell activation markers (11, 22, 23) indicated ineffective T cell response in severe COVID-19 patients. In contrast to previous findings (11), we originally found that ICOS-1 was much better marker for T cell activation correlating with good prognosis, than HLA-DR and CD38. ICOS-1 is critical for the activation and proliferation of T cells, involved in efficient humoral response and suppression of inflammation (47). Moreover, the finding that ICOS-1^-/-^ Т cell fail to produce IL-4 (47), could explain the finding that T cells of mild COVID-19 patients displayed an increased capacity to produce IL-4. A role for Th2 cell-type responses in severe COVID-19 is still unclear (48). An increased Th2 response was described as beneficial in children with COVID-19, due to its potential to reduce exaggerated inflammation (49). However, we observed that severe COVID-19 patients display strongly up-regulated IL-4 production in both CD4^+^ and CD8^+^T cells. These results suggest that the upregulation of Th2 response might be beneficial to some extent, but its further exacerbation could lead to disease progression. On the other hand, IFN-γ-producing CD8^+^ T cells are critical for viral clearance (50). In line with other reports (8, 17) we also found a reduced frequency of IFN-γ-producing CD8^+^ T and Th17 cells in COVID-19 patients, as well as their functional exhaustion marked by increased PD1 expression. Additionally, we showed that mRNA levels of both *T-bet* and *STAT-1* transcription factors, as key regulators of type 1 immune response (51), were also significantly reduced in COVID-19 patients. However, we did not observe direct correlation between *T-bet* expression in PBMC and the percentage of IFN-γ-producing CD8^+^T cells (**Supplementary Figure 3**), suggesting that additional factors could regulate IFN-γ expression in CD8^+^T cells in a *T-bet*-independent manner, such as *Eomes* (52), so this requires further investigations. Lower frequency of Th17 cells corresponded to reduced levels of IL-17A, IL-17F and IL-21 in serum (**Supplementary Figure 2**), presumably because Th17 cells are the dominant producers of these cytokines (53). However, the levels of many cytokines in sera are determined by the number and functional status of cytokine-producing/consuming immune and non-immune cells. Therefore, additional investigations are required to better understand the correlations between serum level of cytokines and the frequency of cytokine-producing cells in COVID-19.

Impaired adaptive immune response indicated dysregulated activation and antigen-presentation. To the best of our knowledge, we showed for the first time that all major populations of APC in blood are affected in COVID-19, and that the expression of key markers contributing to immune cell activation, including MyD88, NF-kB, and RANKL, are also impaired in COVID-19 patients. Additionally, we originally identified osteopontin as an important marker of dysregulated immune response in COVID-19. Sampayo-Escobar et al. (54) showed previously that OPN is partially regulated by IL-1β and increases the susceptibility of epithelial cells for respiratory syncytial virus infection, as well as the spread of the virus. OPN could be involved in similar mechanisms of viral dissemination in COVID-19, so this phenomenon deserved further investigation. Interferon type I-producing pDC are critical for controlling anti-viral response in Dengue virus (39). Preliminary report by Onodi et al. (55) suggested that HCQ might prevent the activation, differentiation and IFN-α production by pDC, thereby preventing an efficient response to SARS-CoV-2. Although both mild and severe COVID-19 patients in our study displayed reduced frequency of pDC in blood, this reduction was significant only for severe COVID-19 patients, suggesting that additional mechanisms might be involved as well, like increased apoptosis in pDC. Preliminary report by Sanchez-Cerrillo et al. (56) suggested that preferential migration of CD1c^+^ DC (DC2) to the lungs, could be associated with dysregulation of CD8^+^T cell activation and poor prognosis for COVID-19 patients. Therefore, it is possible that the lower frequency of CD1c^+^ in severe COVID-19 patients might be related to their increased migration to the lungs, reduced HLA-DR expression, and activation. The frequency of CD141^+^DC (DC1), which are described as the most potent cross-presenting DC critical for an efficient anti-viral response (57), were not significantly changed in COVID-19 patients comparing to healthy donors in our study, and their role in SARS-CoV-2 has yet to be investigated.

Severe patients displayed impaired number and functions of APC along with reduced expression of autophagy-related genes and the expression of IRF-8. IRF-8 was shown essential for HLA-DR expression, migration and cytokine response in a model of human cDC1 (58), as well as for the induction of autophagy and effective innate immune response to *Listeria monocytogenes* infection (27). Moreover, IRF-8 is critical for development of pDC and IFN induction upon TLR/MyD88 signaling (59). Reduced expression of autophagy markers in COVID-19 patients were confirmed by both qPCR analysis of key genes involved in initiation, regulation, nucleation, and elongation steps of autophagy process, as well as Western Blot analysis of LC3 conversion, according to recommendations for autophagy assessment (60). HCQ and CQ were shown to inhibit autophagy by preventing autophagosome/lysosome fusion (61), and concerns were raised about its usage in COVID-19 considering that it could adversely affect acute kidney injury (62). Considering the mechanisms of HCQ actions (61), lower LC3II/LC3I ratio in PBMC of COVID-19 patients was rather a consequence of lower LC3I to LC3II conversion, i.e. lower autophagy flux, than due to increased LC3II degradation in autophagosomes. However, to confirm this hypothesis, direct functional *in vitro* assays, or a group of patients which did not receive HCQ, are necessary in future studies. Although all patients in our study received HCQ, a significantly higher inhibition of autophagy was observed in severe COVID-19 patients, compared to the mild patients. These results suggest that possibly additional mechanisms contribute to autophagy inhibition in COVID-19. In line with this, a sustained over-production of IL-6 was shown to block autophagy by supporting Beclin-1/Mcl-1 interaction, and vice versa, the induction of autophagy could counteract the effects of IL-6 (63, 64). A strong negative correlation between the sera levels of IL-6 and autophagy genes expression in PBMC was observed in our study for the first time (**Supplementary Figure 3**), supporting the hypothesis on direct pathogenic effects of IL-6 on autophagy and antigen presentation.

Previous data suggested that the most important sources of IL-6 in COVID-19 patients were circulating monocytes (17, 65). One of the most important findings of this study was that COVID-19 patients display strong expansion of PMN-MDSC and Mo-MDSC, the latter of which contribute mostly to IL-6 production in severe COVID-19 patients. Although previous reports suggested that MDSC could play important roles in COVID-19 (66), no detailed characterization of MDSC subtypes and their functions in COVID-19 has been reported. Besides GM-CSF, which was described as an important pathogenic factor in COVID-19 (16), IL-6 is critical for the induction of Mo-MDSC from peripheral monocytes (19), and its role in PMN-MDSC induction was also demonstrated (30). Curiously enough, Tocilizumab therapy in our study did not affect the percentage of MDSC subsets nor the sera IL-6 levels. This result could be a consequence of premature sampling of cells from COVID-19 patients receiving the therapy, but also the implication of additional mechanisms involved in MDSC expansion. Besides IL-6, IRF-8 was described as a key negative regulator of MDSC, as IRF8^-/-^ mice display marked accumulation of MDSC in a tumor model (67). These authors also showed that the blockage of STAT-mediated IRF-8 downregulation could prevent MDSC accumulation and dysregulation of the immune response in tumor. It was shown that SARS-CoV-2 may directly block interferon *via* ORF3b (68). Our results indicated for the first time that one possible downstream effect of this blockage is reduced IRF-8 expression, potentially leading to reduction of autophagy, impaired HLA-DR expression and pDC functions, followed by MDSC expansion. However, additional investigations are necessary to clarify the roles of IRF-8 expression in specific subsets of PBMC, which could be beneficial for developing novel therapeutic approaches modulating IRF8-dependent pathways in COVID-19.

MDSC display abundant immune suppressive mechanisms including IL-10, TGF-β, Arg-1, IDO-1, ILT3, COX-2, PD1L, and others (69), all of which were clearly increased in COVID-19 patients in this study. NADPH-oxidase dependent immune suppression is the major mechanism of PMN-MDSC (69). Oddly enough, we detected a lower expression of NOX-1 mRNA, even though PMN-MDSC expanded significantly in these patients. The significance of this finding is still not clear. It is possible that NOX-1 expressing PMN-MDSC preferentially emigrated to the lungs causing ROS-mediated damage of the tissue, as described in severe COVID-19 (70). We also found that Arg-1 expression in PBMC correlated strongly with serum levels of urea, which is expected considering the role of this enzyme in urea generation (71). Therefore, the measurement of serum urea could be one of the indicators for MDSC expansion in COVID-19. Besides direct suppressive effects on immune cells, MDSC can expand different subsets of regulatory cells in antigen dependent and antigen-independent manner (69). Here we showed for the first time that severe COVID-19 patients have increased accumulation of different Treg subsets, including conventional Tregs, Tr-1 cells, suppressor CD8^+^ T cells, TGF-β-producing Th3 cells and Bregs. Using a model of monocyte derived Mo-MDSC we showed previously that IDO-1, ILT-3, and ILT-4 are critically involved in the expansion of conventional CD4^+^ Tregs and Tr-1 cells (19). It is highly possible that similar mechanisms mediate the expansion of regulatory lymphocytes by MDSC in severe COVID-19 patients, and hierarchical cluster analysis (**Supplementary Figure 3**) supports this hypothesis. These findings could explain the phenomenon of immune paralysis observed in COVID-19 patients. Therefore, the therapy targeting immunosuppressive mechanisms mediated by MDSC in COVID-19, might provide great clinical benefits. Previous studies showed that 5-fluorouracil (5-FU) applied in small doses, selectively kill MDSC and enhances Th1 cells response (33). In line with this, in an *in vitro* pilot study we showed that 5-FU may lower the burden of MDSC in severe COVID-19 patients, casing a lowered production of IL-10, IL-8, IL-17, and Th2 cytokines, while increasing the production of IFN-γ and IL-2. Therefore, shifting the immunological balance toward increased Th1 immune response and lymphocytes proliferation could provide large clinical benefit in COVID-19 patients. It was suggested that 5-FU in combination with deoxyribonucleosides and deoxyribose could exhibit antiviral effects in COVID-19 therapy (72). The first experimental data obtained in this study encourage further testing for off-label application of 5-FU in the treatment of severe COVID-19 patients.

Cumulatively, by extensive analyses of ~160 immunological and clinical parameters we identified previously unrecognized immunological mechanisms of dysregulated immune response which could strongly predict for COVID-19 severity and represent potential therapeutic targets. Namely, we observed that SARS-CoV-2 infected patients displayed reduced expression of autophagy-related genes, altered differentiation and functions of antigen presenting cells, and expansion of MDSC, possibly *via* IL-6 and IRF-8-mediated mechanisms, all of which correlated with inappropriate activation and functions of T cells in these patients. Thereby, MDSC were probably the dominant pathogenic factors in COVID-19, driving both exaggerated inflammation and, together with regulatory lymphocytes, immune paralysis in severe patients. Additional investigations are necessary, such as analyses of gene expression in specific immune cell subsets of COVID-19 patients followed by appropriate functional assays, to develop targeted therapies aimed to reduce MDSC-mediated immunopathologic effects and restore efficient T cell responses in patients with severe COVID-19.

## Data Availability Statement

The original contributions presented in the study are included in the article/**Supplementary Material**. Further inquiries can be directed to the corresponding author.

## Ethics Statement

The studies involving human participants were reviewed and approved by Ethical Boards of the KBC Zemun. The patients/participants provided their written informed consent to participate in this study.

## Author Contributions

ST, JĐ, and MČ designed research study. ST, JĐ, NI, AG-M, MD, and MB conducted experiments. ST, JĐ, DeS, NI, AG-M, MD, DR, MB, and DrS collected data. DR and JĐ performed metadata analysis and graphical illustrations. ST, JĐ, DeS, NI, MD, DR, and MB analyzed data. ST, DeS, NM, RT, DM, and DrS collected peripheral blood samples and acquired informed consents. ST and JĐ wrote the manuscript. DM, DrS, and MČ provided advice. All authors contributed to the article and approved the submitted version.

## Funding

The study was supported by the Ministry of Education, Science and Technological Development, Republic of Serbia (Contract No. 451-03-68/2020-14/200019, Contract No. 451-03-68/2020-14/200042, and Grant No. 451-03-921/2020-14/6 “Immunological aspects of SARS-CoV-2 infections”) and Serbian Academy of Sciences and Arts (Grant No. F31). The funders had no role in study design, data collection and analysis, decision to publish, or preparation of the manuscript

## Conflict of Interest

The authors declare that the research was conducted in the absence of any commercial or financial relationships that could be construed as a potential conflict of interest.
