# Supplementary material for: Reduced Expression of Autophagy Markers and Expansion of Myeloid-Derived Suppressor Cells Correlate With Poor T Cell Response in Severe COVID-19 Patients
Source: Front Immunol. 2021 Feb 22;12:614599. doi: 10.3389/fimmu.2021.614599 (PMC7937809; doi:10.3389/fimmu.2021.614599)
Supplement: Supplementary file 1 [file DataSheet_1.docx]

Supplementary Material

# Supplementary Figures and Tables

## Supplementary Figures


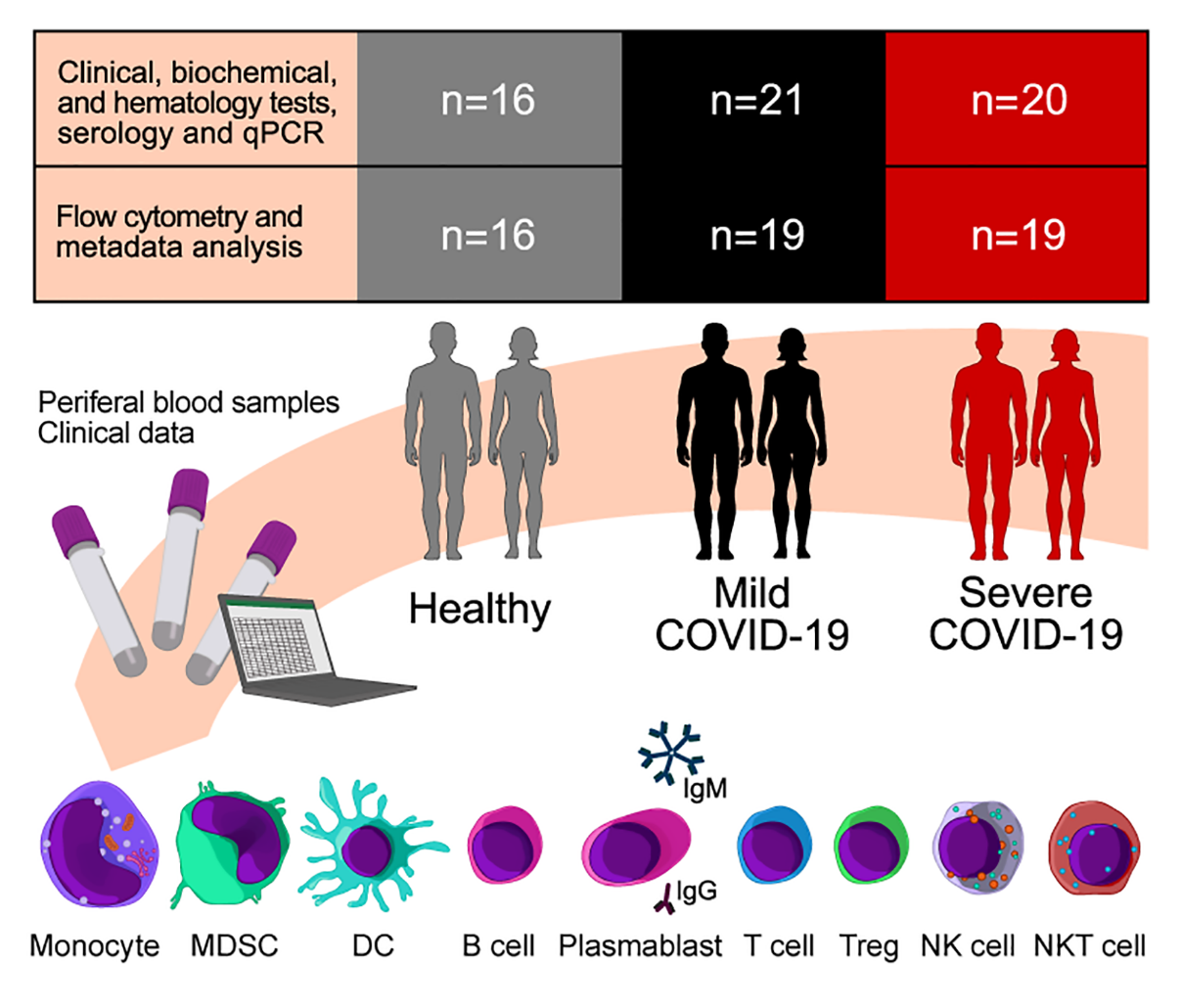


**Supplement Figure 1.** Experimental design of the study. Samples of peripheral blood and sera were collected form 16 healthy donors and 41 COVID-19 patients, of which 20 patients showed severe COVID-19 pathology (red) and 21 patients displayed mild COVID-19 symptoms. Clinical, biochemical, hematological data, as well as serology, qPCR, flow cytometry and metadata analyses were performed on indicated number of donors to evaluate the functions of key innate and adaptive immune cells of humoral and cellular immune response.


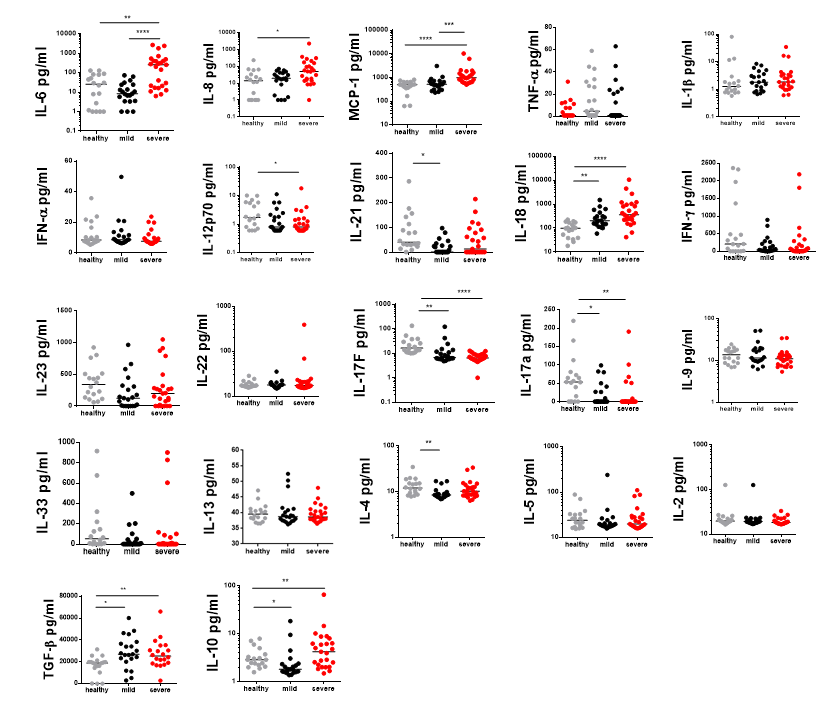


**Supplement Figure 2.** Sera levels of cytokines from COVID-19 patients and healthy donors. The levels all cytokines in samples of sera were quantified by flow cytometry using LEGENDplex system, except the levels of TGF-β, IL-33 and IL-1β which were quantified by Duo Set ELISA tests. The concentrations of cytokines were calculated from standard curves. For the levels of cytokines below the limit of detection (LOD) specified for each cytokine by manufacturer, the LOD values were appointed to these samples in order to include each donor specimen in the statistical analysis. The summarized results form 16 healthy donors (gray dots), 21 mild (black dots) and 20 severe patients (red dots) are shown with median values. *p<0.05, **p<0.01, ***p<0.005, ****p<0.001 as indicated (Kruskal-Wallis test with Dunn’s post-test).


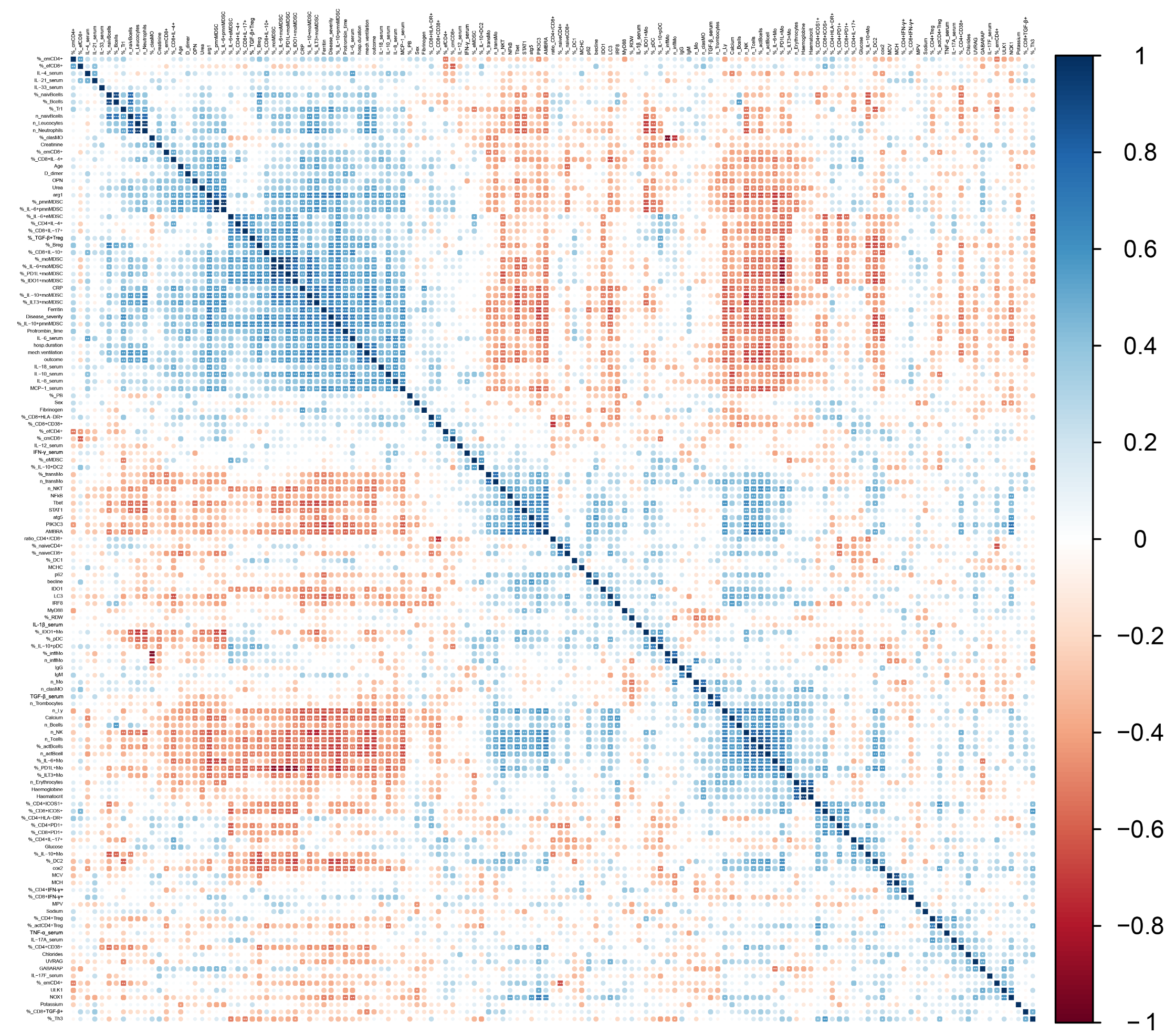


**Supplement Figure 3.** Correlations of clinical, laboratory and immunological parameters in COVID-19 patients. Spearman’s correlation matrix and hierarchical clustering of 135 tested features in COVID-19 patients (19 mild and 19 severe). The color and size of the circles represent the correlation coefficient. Asterisks indicate significance levels for each comparison, at FDR below 0.05 (*), 0.01 (**), and 0.001 (***). n_, number; Ly- lymphocytes; Mo- monocytes, cm-central memory, ef- effector, em- effector memory, act-activated, clas-classical.


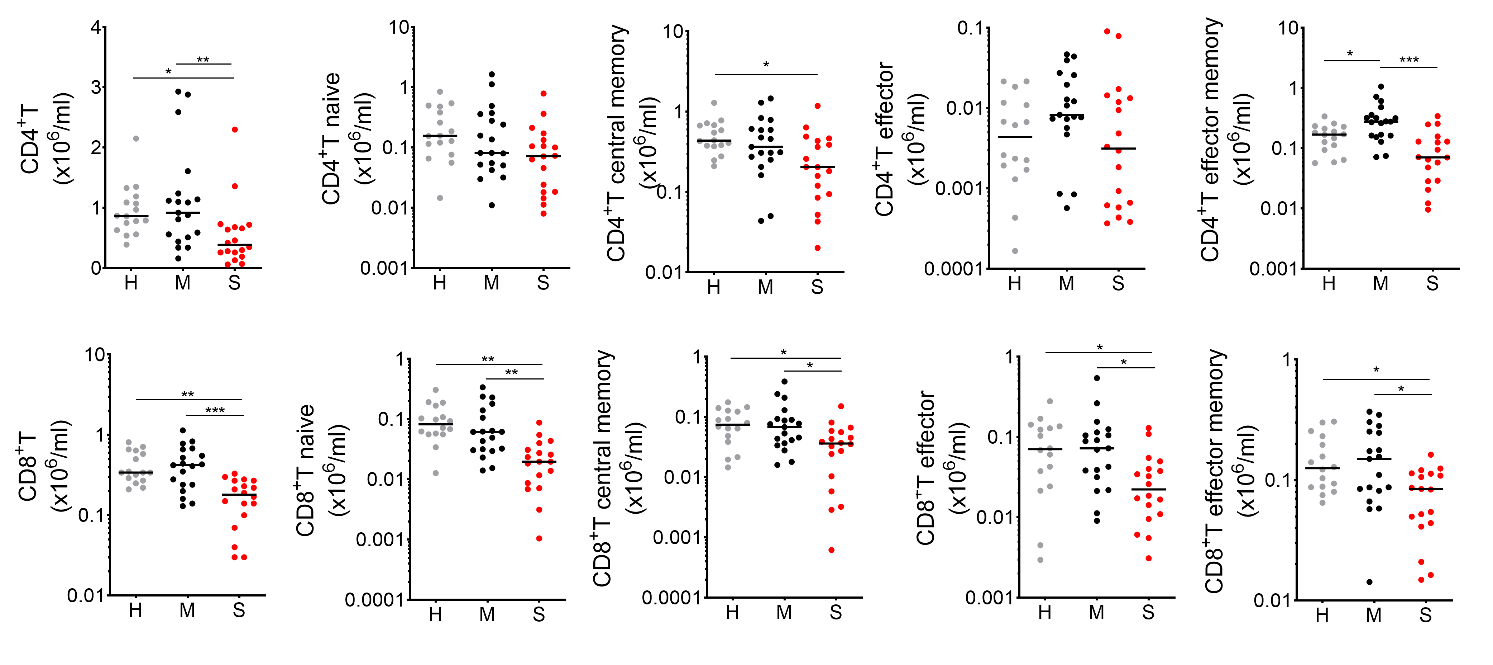


**Supplementary Figure 4.** The number of T cell subsets in COVID-19 patients and healthy donors. The total number of naïve (CD62L^+^CD45^+^), effector (CD62L^-^CD45RA^+^), central memory (CD62L^+^CD45RA^-^) and effector memory (CD62L^-^CD45RA^-^) CD4 and CD8 T cells was calculated based on flow cytometry data and the number T cells (Figure 1A, B, Figure 2A, B). The results for each donor in the groups of healthy donors (H, n=16), mild (M, n=19) and severe (S, n=19) patients, and the corresponding medians are shown. *p<0.05, **p<0.01, ***p<0.005, ****p<0.001 as indicated (Kruskal-Wallis test with Dunn’s post-test).


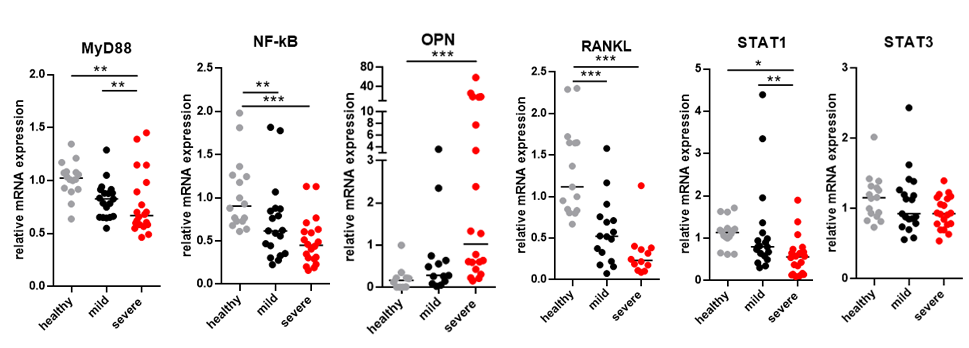


**Supplement Figure 5.** Relative mRNA expression of signal transducing and transcription factors in COVID-19 and healthy donors. Relative mRNA expressions of MyD88, NF-kB, OPN, RANKL, STAT-1 and STAT-3 were analyzed in PBMC of 16 healthy donors (grey dots), 19 mild (black dots) and 19 severe (red dots) COVID-19 patients by qPCR. *p<0.05, **p<0.01, ***p<0.005 as indicated (Kruskal-Wallis test with Dunn’s post-test).


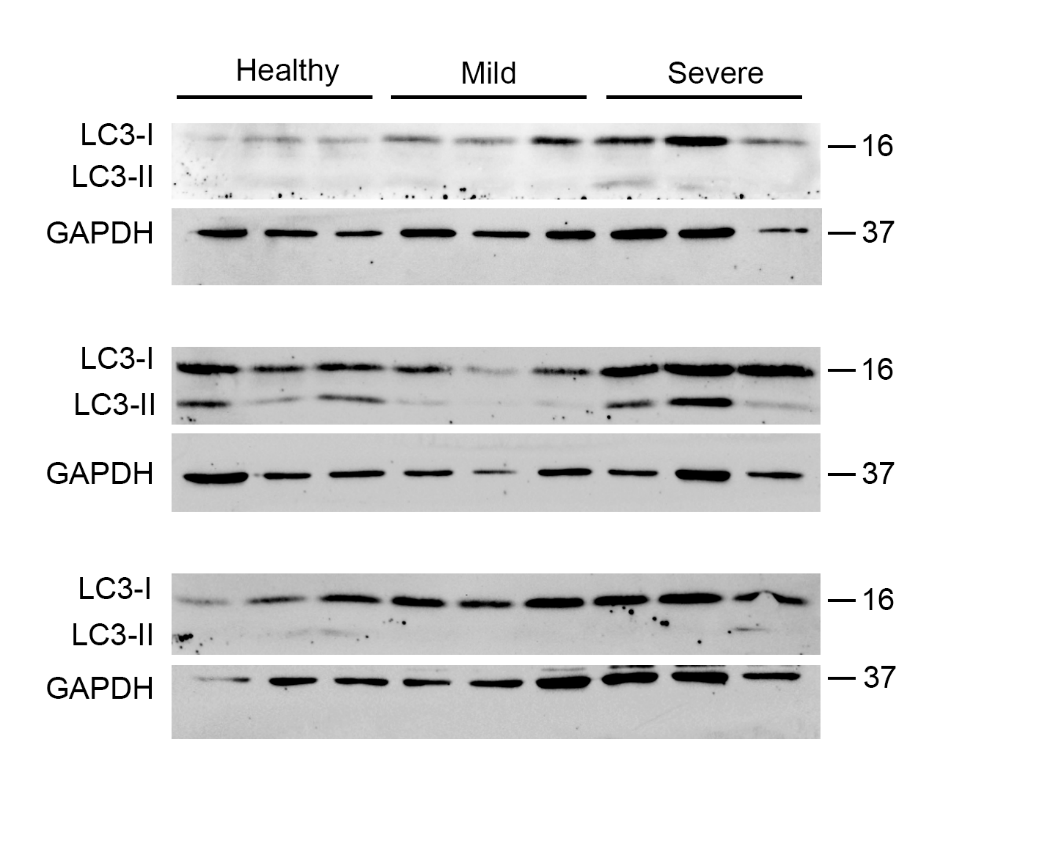


**Supplement Figure 6.** Analysis of LC3I and LC3II expression ratio in healthy donors and COVID-19 patients. Expression of LC3I, LC3II and GAPDH from totally 9 healthy donors, 9 mild patients and 9 severe patients were analyzed. Western blot analysis of LC3I (16kDa), LC3II and GAPHD (37kDa) are shown, wherein each blot in a column of a row was prepared from PBMC of individual donor.


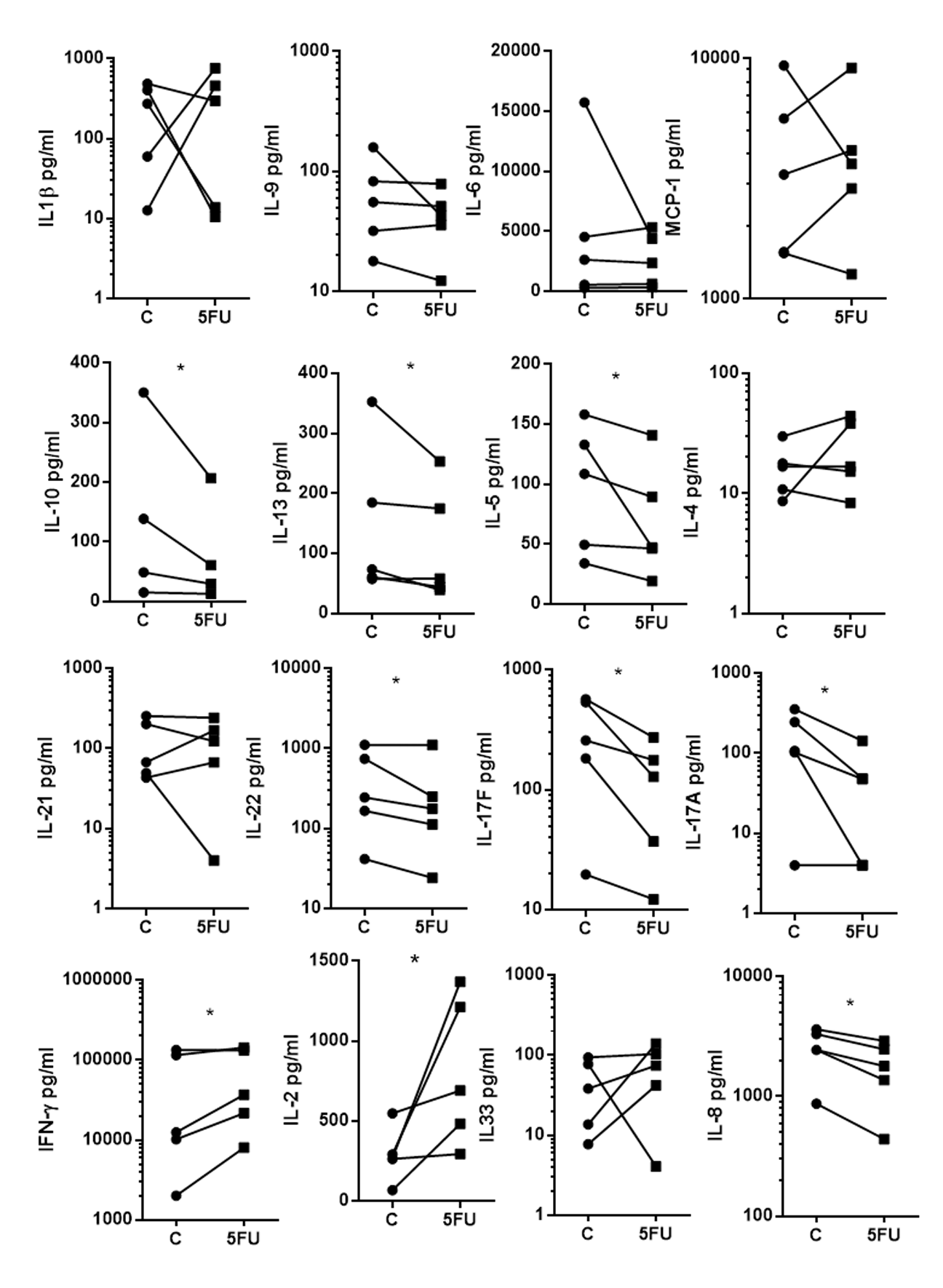


**Supplement Figure 7.** Effects of 5-fluorouracil on cytokines production in monocytic cells/T cell co-cultures. Indicated cytokine levels were determined by LEGENDplex system in supernatants collected from co-cultures of MACS purified monocytic cells (0.25x10^5^ cells/well) from 5 severe patients and allogeneic MACS purified T cells (1x10^5^) from a healthy donor in the presence of CD3/CD28 beads for 3 days. 5-fluorouracil (5-FU, 5µM) was added to test cultures at day 0, whereas control cultures (C) received equivalent volume of PBS. Dots connected by lines originate from co-cultures with the same donor of monocytic cells *p<0.05 (Student T-test).
