# Supplementary material for: Reduced Expression of Autophagy Markers and Expansion of Myeloid-Derived Suppressor Cells Correlate With Poor T Cell Response in Severe COVID-19 Patients
Source: Front Immunol. 2021 Feb 22;12:614599. doi: 10.3389/fimmu.2021.614599 (PMC7937809; doi:10.3389/fimmu.2021.614599)
Supplement: Supplementary file 2 [file DataSheet_2.pdf]

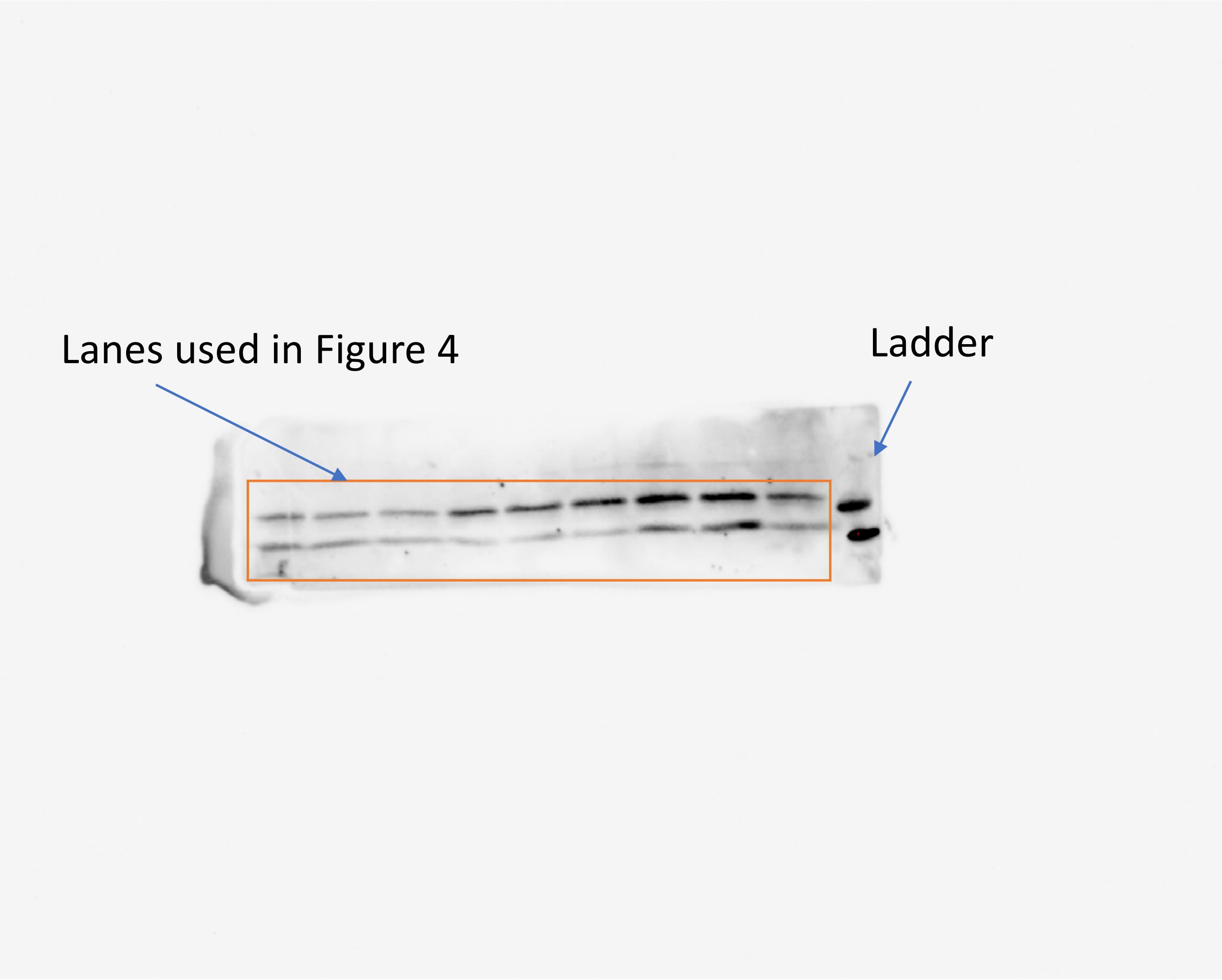

Full unedited gel for Figure 4B.
Staining was performed with anti-human LC3 Ab (1:2000, Invitrogen, PA1-16930) followed by goat anti-rabbit HRP-conjugated IgG H&L Ab (1:10000 Thermo Scientific)

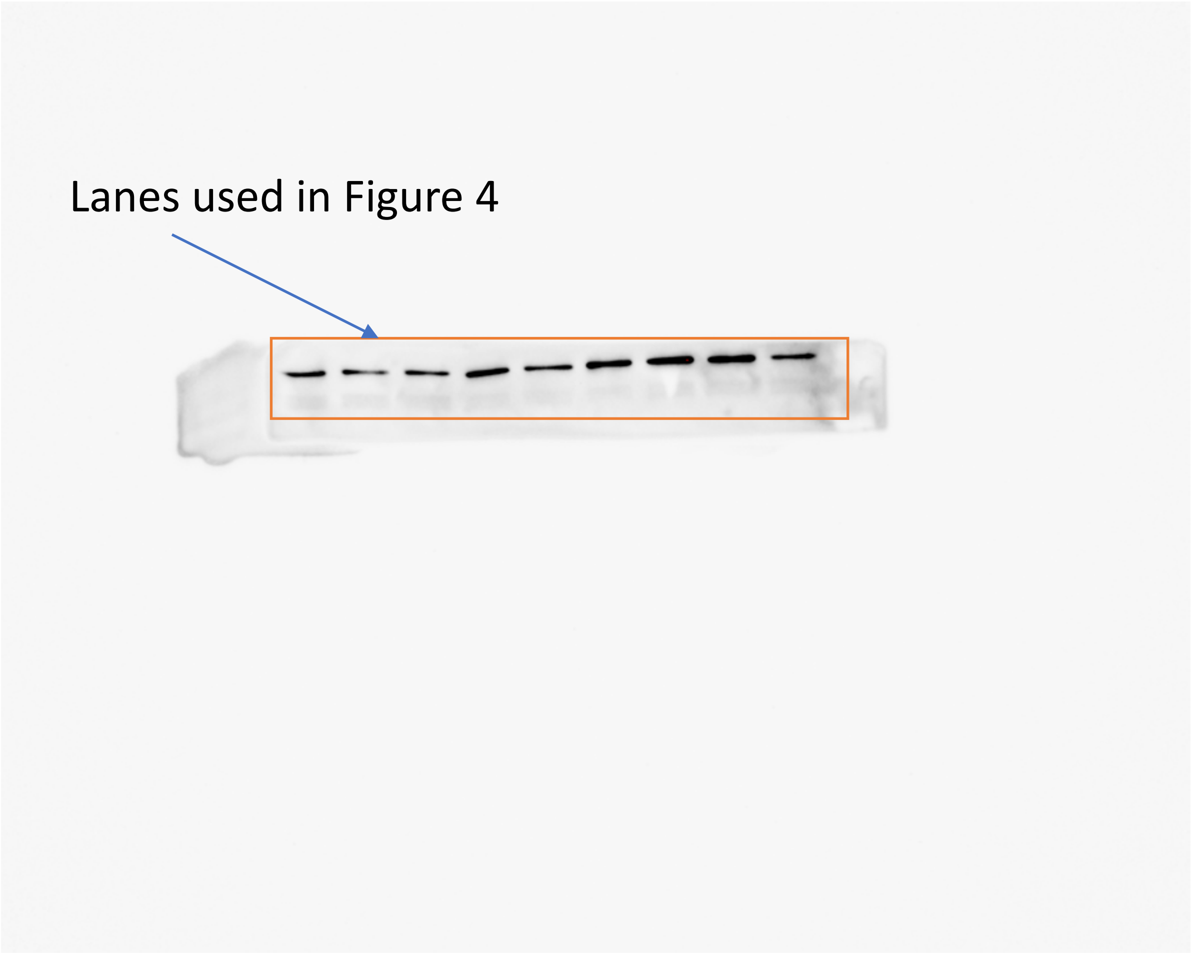

Full unedited gel for Figure 4B.
Staining was performed with anti-human GAPDH Ab (1:2000, Abcam, ab9484) followed by goat anti-mouse HRP-conjugated IgG H&L Ab (1:10000 Amersham Biosciences)

Full unedited gels for Supplement Figure 5.

Lanes used in
Supplement Figure 5
First row

Lanes used in
Supplement Figure 5
Second row

Lanes used in
Supplement Figure 5
Third row

LC3 staining as indicated on slide 1

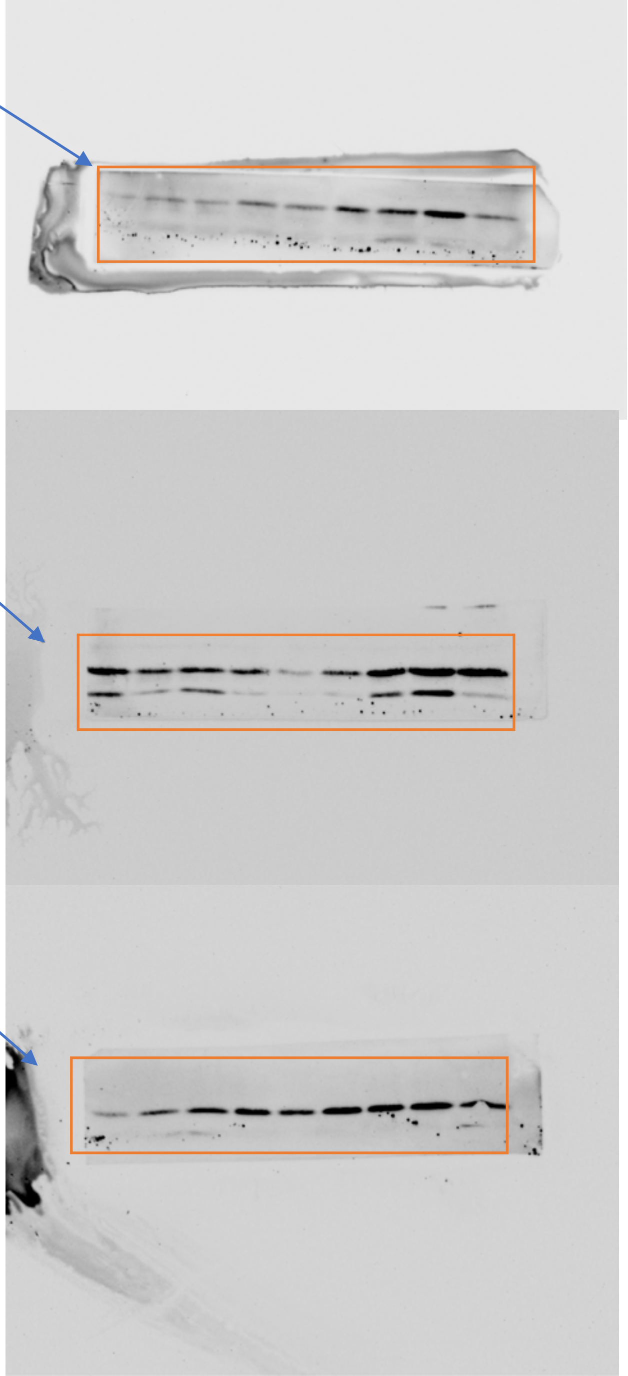

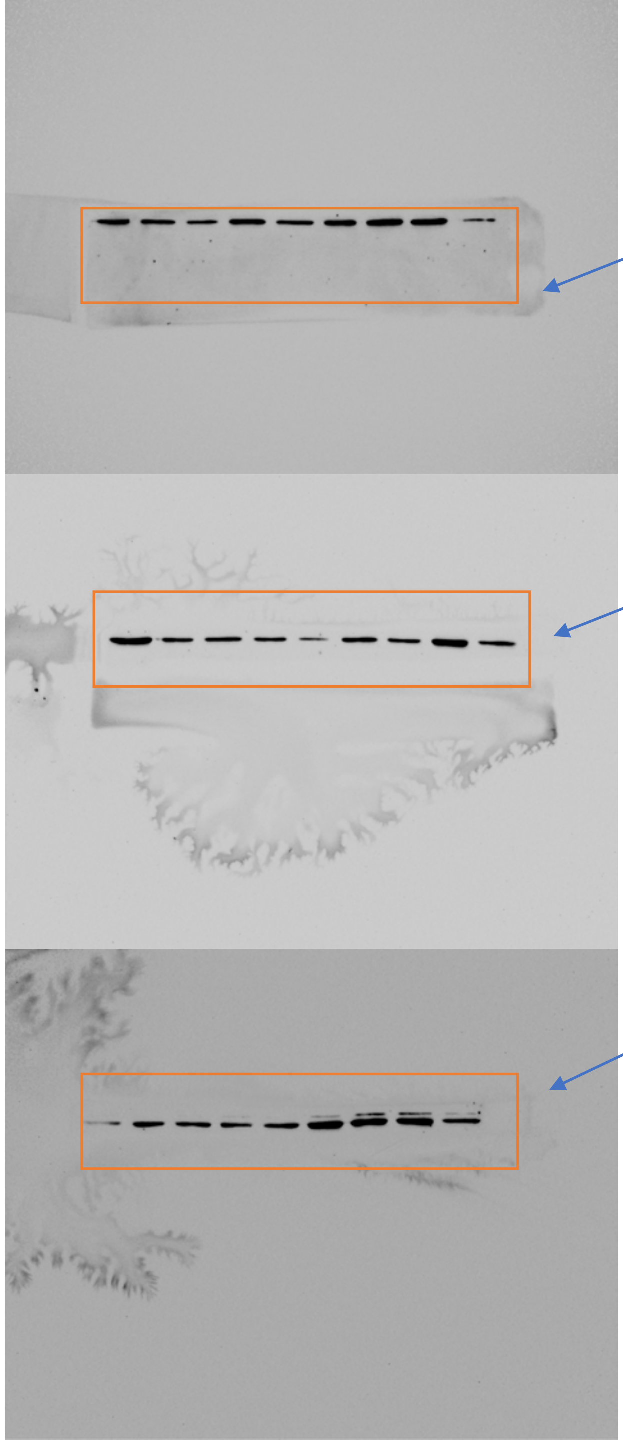

Lanes used in
Supplement Figure 5
First row

Lanes used in
Supplement Figure 5
Second row

Lanes used in
Supplement Figure 5
Third row

GAPDH staining as indicated on slide 2
