# Supplementary material for: Reduced Expression of Autophagy Markers and Expansion of Myeloid-Derived Suppressor Cells Correlate With Poor T Cell Response in Severe COVID-19 Patients
Source: Front Immunol. 2021 Feb 22;12:614599. doi: 10.3389/fimmu.2021.614599 (PMC7937809; doi:10.3389/fimmu.2021.614599)
Supplement: Supplementary file 3 [file Table_1.docx]

| **Supplementary Table 1. List of monoclonal anti-human antibodies used for flow cytometry** | | |
| --- | --- | --- |
| **Monoclonal antibody to: fluorochrome** | **Clone** | **Company** |
| CD14:FITC | HCD14 | Biolegend |
| CD11b:PE-Cy7 | ICRF44 | eBioscience |
| CD16:PE | 3G8 | Biolegend |
| CD15:PerCP-Cy5.5 | HI98 | Biolegend |
| CCR2:PerCP-Cy5.5 | K036C2 | Bioleged |
| HLA-DR:APC-Cy7 or FITC | L243 | Biolegend |
| HLA-DR:PerCP | REA805 | Miltenyi Biotec |
| CD45: APC | 2D1 | Biolegend |
| CD3:PE | UCHT1 | Elabscience |
| CD62L:FITC | DREG-56 | Biolegend |
| CD4:PerCP-Cy5.5 | MEM-241 | Exbio |
| CD8:PE-Cy7 | MEM-31 | Exbio |
| ILT3:PE-Cy7 | ZM4.1 | Biolegend |
| PD1L:APC or PE | 29E.2A3 | Biolegend |
| PD1:APC or APC-Cy7 | A17188B | Biolegend |
| IDO-1:APC | 700838 | R&D Systems |
| IL-6:PE | MQ2-13A5 | Biolegend |
| IL10:PE | JES3-19F1 | BD Pharmigen |
| IL10:APC | 3F9 | Biolegend |
| IL-4:PE or PerCP-Cy5.5 | 8D4-8 | Biolegend |
| IFN-γ: FITC | 4S.B3 | Thermofisher |
| IFN-γ: APC | B27 | Elabscience |
| IL-17A:Alexa fluor 488 | BL168 | Biolegend |
| IL-17A:APC | CZ8-23G1 | Miltenyi Biotec |
| TGF-β1:APC or PE | TW4-2F8 | Biolegend |
| CD25: PE-Cy7 | MEM-181 | Exbio |
| FoxP3:FITC | 206D | Biolegend |
| CD127:PE | A019D5 | Biolegend |
| ICOS-1:APC-Cy7 | REA192 | Miltenyi Biotec |
| CD19:PerCP-Cy5.5 | 4G7 | Miltenyi Biotec |
| CD38:APC-Cy7 | HB-7 | Biolegend |
| CD66b:APC | G10F5 | Biolegend |
| CD56:FITC | 5.1H11 | Biolegend |
| CD1c:FITC | AD5-8E7 | Miltenyi Biotec |
| CD141:PE | AD5-14H12 | Miltenyi Biotec |
| CD123:biotin | 9F5 | BD Pharmigen |
| CD40:FITC | REA733 | Miltenyi Biotec |
| CD40:APC | LOB7/6 | BioRad |
| IgM:Biotin | MHM-88 | Biolegend |
