# Supplementary material for: Reduced Expression of Autophagy Markers and Expansion of Myeloid-Derived Suppressor Cells Correlate With Poor T Cell Response in Severe COVID-19 Patients
Source: Front Immunol. 2021 Feb 22;12:614599. doi: 10.3389/fimmu.2021.614599 (PMC7937809; doi:10.3389/fimmu.2021.614599)
Supplement: Supplementary file 4 [file Table_2.docx]

**Table S2. List of primers used in this study.**

| **Primer name** | **Primer sequence 5’–3’** | **Reference** |
| --- | --- | --- |
| AMBRA1_F | GGTGGGAGGAGAGGGGATAG | This work |
| AMBRA1_R | CGAGGGGCATGTCATCATTT |  |
| ULK1_F | TTTTGTTTCTCCGTTGGGGC | This work |
| ULK1_R | ACTCTTCCCGGGCTGCTAAT |  |
| UVRAG_F | AGGAAGGAGTGCACTGCAAA | This work |
| UVRAG_R | AGGCAACTTGACACCGCATA |  |
| GABARAP_F | CCCTCGTCCCGCTGATTTTA | This work |
| GABARAP_R | ATCCCTCCAGCTTGTACCCA |  |
| PIK3C3_F | GCTGTCCTGGAAGACCCAAT | This work |
| PIK3C3_R | TTCTCACTGGCAAGGCCAAA |  |
| MAP1LC3B_F | TTCAGGTTCACAAAACCCGC | This work |
| MAP1LC3B_R | TCTCACACAGCCCGTTTACC |  |
| BECN1_F | CTGGGACAACAAGTTTGACCAT | Liu et al., 2014 |
| BECN1_R | GCTCCTCAGAGTTAAACTGGGTT |  |
| ATG5_F | CACAAGCAACTCTGGATGGGATTG | He et al., 2013 |
| ATG5_R | GCAGCCAC GGACGAAACAG |  |
| SQSTM1_F | GCCAGAGGAACAGATGGAGT | Sahani et al., 2014 |
| SQSTM1_R | TCCGATTCTG GCATCTGTAG |  |
| STAT1_F | ACTGCTGTCTATGTGGCTGT | This work |
| STAT1_R | GGAGGCCTCAGATTGTATGC |  |
| STAT3_F | TCTGCCGGAGAAACAGTTGG | This work |
| STAT3_R | ATCCAAGGGGCCAGAAACTG |  |
| IRF8_F | CGGAGTCCCTGAATCTGATGT | This work |
| IRF8_R | GGATCCGGAACATGCTCTTCT |  |
| NFKB1_F | TGAGAGTCACTTGATGTGCCA | This work |
| NFKB1_R | GCAAAATGCAGAAAAGGGGGA |  |
| PTGS2_F | CCGGGTACAATCGCACTTAT | Han et al., 2020 |
| PTGS2_R | GGCGCTCAGCCATACAG |  |
| ARG1_F | GTGGAAACTTGCATGGACAAC | Kong et al., 2017 |
| ARG1_R | AATCCTGGCACATCGGGAATC |  |
| NOX1_F | GCCATGACTGTAGCAAGGCT | This work |
| NOX_R | AAATCTTCTGTGGGGGTGGG |  |
| RANKL_F | TTGAGCCCCAGTTTTTGGAGTG | This work |
| RANKL_R | ACATCTCCCACTGGCAGGTAAAT |  |
| OPN_F | CATACAAGGCCATCCCCGTT | This work |
| OPN_R | GGGTTTCAGCACTCTGGTCA |  |
| MyD88_F | CAGTTGCCGGATCTCCAAGT | Popovic et al., 2019 |
| MyD88_R | GTCTCCTCCACATCCTCCCT |  |
| PD1_F | CAGTTCCAAACCCTGGTGGT | Andreev et al., 2016 |
| PD1_R | GGCTCCTATTGTCCCTCGTG |  |
| IDO-1_F | GGGACACTTTGCTAAAGGCG | Folgiero et al., 2014 |
| IDO-1_R | GTCTGATAGCTGGGGGTTGC |  |
| T-bet_F | TGGTGTGGACTGAGATTGCC | This work |
| T-bet_R | CCACTGGAAGGATAGGGGGA |  |
| GAPDH_F | GTGAAGGTCGGAGTCAACG | Kretschmer et al., 2015 |
| GAPDH_R | TGAGGTCAATGAAGGGGTC |  |

Liu, K., Shi, Y., Guo, X., Wang, S., Ouyang, Y., Hao, M., et al. (2014). CHOP mediates ASPP2-induced autophagic apoptosis in hepatoma cells by releasing Beclin-1 from Bcl-2 and inducing nuclear translocation of Bcl-2. Cell Death Dis. 5(7):e1323.

He, Z., Liu, H., Agostini, M., Yousefi, S., Perren, A., Tschan, M.P., et al. (2013). p73 regulates autophagy and hepatocellular lipid metabolism through a transcriptional activation of the ATG5 gene. Cell Death Differ. 20(10), 1415–24.

Sahani, M.H., Itakura, E., and Mizushima, N. (2014). Expression of the autophagy substrate SQSTM1/p62 is restored during prolonged starvation depending on transcriptional upregulation and autophagy-derived amino acids. Autophagy. 10, 431–41.

Kong D, Li J, Shen Y, et al. Niacin Promotes Cardiac Healing after Myocardial Infarction through Activation of the Myeloid Prostaglandin D2 Receptor Subtype 1. J Pharmacol Exp Ther. 2017;360(3):435-444. doi:10.1124/jpet.116.238261

Han L, Fang S, Li G, Wang M, Yu R. Total flavonoids suppress lung cancer growth via the COX-2-mediated Wnt/β-catenin signaling pathway. Oncol Lett. 2020;19(3):1824-1830. doi:10.3892/ol.2020.11271

Andreev K, Trufa DI, Siegemund R, et al. Impaired T-bet-pSTAT1α and perforin-mediated immune responses in the tumoral region of lung adenocarcinoma [published correction appears in Br J Cancer. 2016 Oct 25;115(9):e11]. Br J Cancer. 2015;113(6):902-913. doi:10.1038/bjc.2015.25

Kretschmer I, Freudenberger T, Twarock S, Fischer JW. Synergistic effect of targeting the epidermal growth factor receptor and hyaluronan synthesis in oesophageal squamous cell carcinoma cells. Br J Pharmacol. 2015;172(18):4560-4574. doi:10.1111/bph.13240

Popović N, Djokić J, Brdarić E, Dinić M, Terzić-Vidojević A, Golić N and Veljović K (2019) The Influence of Heat-Killed Enterococcus faecium BGPAS1-3 on the Tight Junction Protein Expression and Immune Function in Differentiated Caco-2 Cells Infected With Listeria monocytogenes ATCC 19111. Front. Microbiol. 10:412. doi: 10.3389/fmicb.2019.00412

Folgiero V, Goffredo BM, Filippini P, et al. Indoleamine 2,3-dioxygenase 1 (IDO1) activity in leukemia blasts correlates with poor outcome in childhood acute myeloid leukemia. Oncotarget 2014;5:2052-64.
