# Supplementary material for: Reduced Expression of Autophagy Markers and Expansion of Myeloid-Derived Suppressor Cells Correlate With Poor T Cell Response in Severe COVID-19 Patients
Source: Front Immunol. 2021 Feb 22;12:614599. doi: 10.3389/fimmu.2021.614599 (PMC7937809; doi:10.3389/fimmu.2021.614599)
Supplement: Supplementary file 5 [file Table_3.docx]

| **Supplementary Table 3. Clinical parameters, therapy and follow-up of COVID-19 patients involved in the study** | | | | | | | | | | |
| --- | --- | --- | --- | --- | --- | --- | --- | --- | --- | --- |
| **Patient** | **Gender** | **Age** | **Symptoms on day of analysis** | **Lung imaging finding** | **Comorbidity** | **Complications** | **Therapy before the day of analysis** | **Mechanical ventilation during the course of disease** | **Condition on day of analysis** | **Follow-up (day from analysis)** |
| A1 | M | 34 | Malaise | CXR: Normal finding | / | / | HCQ | No | Hospitalized (D05) | Clinically recovered, discharged home - D11 |
| A2 | F | 33 | Dyspnoea, cough | CXR: Incipient pneumonia - Unillateral | / | / | HCQ | No | Hospitalized (D05) | Clinically recovered, discharged home - D12 |
| A3 | F | 39 | Fever, cough | CXR: Incipient pneumonia - Unillateral | / | / | HCQ | No | Hospitalized (D07) | Clinically recovered, discharged home - D13 |
| A4 | F | 22 | Fever, diarrhea, headache | CXR: Incipient pneumonia - Billateral | / | / | HCQ | No | Hospitalized (D08) | Clinically recovered, discharged home - D10 |
| A5 | F | 57 | Fever, anosmia | CXR: Incipient pneumonia - Billateral | CVD | / | HCQ | No | Hospitalized (D15) | Clinically recovered, discharged home - D30 |
| A6 | M | 68 | Malaise, pain in throat | CXR: Incipient pneumonia - Billateral | CVD, TBC pulmonum | / | HCQ | No | Hospitalized (D15) | Clinically recovered, discharged home - D19 |
| A7 | M | 42 | Malaise | CXR: Incipient pneumonia - Billateral | / | / | HCQ | No | Hospitalized (D11) | Clinically recovered, discharged home - D13 |
| A8 | M | 28 | Malaise, cough | CXR: Incipient pneumonia – Billateral | / | / | HCQ | No | Hospitalized (D05) | Clinically recovered, discharged home - D09 |
| A9 | M | 51 | Cough | CXR: Incipient pneumonia - Billateral | / | / | HCQ | No | Hospitalized (D05) | Clinically recovered, discharged home - D09 |
| A10 | F | 36 | Nasal congestion | CXR: Incipient pneumonia - Billateral | / | / | HCQ | No | Hospitalized (D06) | Clinically recovered, discharged home - D10 |
| A11 | F | 55 | Malaise | CXR: Incipient pneumonia - Billateral | / | / | HCQ, LMWH | No | Hospitalized (D06) | Clinically recovered, discharged home - D13 |
| A12 | F | 72 | Malaise, cough | CXR: Pneumonia – Left | S, psychiatric patient | / | HCQ, LMWH | No | Hospitalized (D05) | Clinically recovered, discharged home - D10 |
| A13 | M | 57 | Malaise, fever | CXR: Pneumonia - Billateral | M (St. post op et RT) St post PTE a.m. III, St post DVT l. sin. a.m. III | / | C, HCQ, LMWH | No | Hospitalized (D15) | Clinically recovered, discharged home - D29 |
| A14 | M | 70 | Malaise, dyspnoea, cough | CXR: Incipient pneumonia - Billateral | DBT | / | HCQ, LMWH | No | Hospitalized (D06) | Clinically recovered, discharged home - D10 |
| A16 | F | 50 | Malaise, fever, cough | CXR: Incipient pneumonia - Billateral | H | / | HCQ, LMWH | No | Hospitalized (D05) | Clinically recovered, discharged home - D14 |
| A17 | M | 54 | Chst pain, malaise, fever, cough, | CXR: Pneumonia - Billateral | / | / | C, HCQ, LMWH | No | Hospitalized (D05) | Clinically recovered, discharged home - D11 |
| A18 | M | 76 | Malaise, fever | CXR: Incipient pneumonia - Billateral | H | / | HCQ, LMWH | No | Hospitalized (D05) | Clinically recovered, discharged home - D10 |
| A20 | M | 66 | / | CXR: Incipient pneumonia - Billateral | M, H, DBT | / | C, HCQ, LMWH | No | Hospitalized (D06) | Clinically recovered, discharged to another clinic - D21 |
| A21 | M | 61 | / | CXR: Normal finding | Melaena | / | / | No | Hospitalized (D05) | Clinically recovered, discharged home - D08 |
| A22 | F | 78 | Cough | CXR: Normal finding CT: PTE | Crural varicosities, AF, DYS | / | HCQ, LMWH | No | Hospitalized (D10) | Clinically recovered, discharged to another clinic - D16 |
| B1 | M | 31 | Malaise, fever | CXR: Normal finding | / | / | Cefixime, HCQ, LMWH | No | Hospitalized (D10) | Clinically recovered, discharged home - D20 |
| B2 | M | 53 | Diarrhoea, malaise, fever, cough, nausea | CXR: Pneumonia - Billateral | Duodenal ulcer in the past | Left pneumothorax | Mem, HCQ, LMWH, L/R | Yes - IV | Hospitalized (D20) | Clinically recovered, discharged home - D20 |
| B3 | M | 63 | Malaise, fever | CXR: Pneumonia - Billateral | M (St. post op et RT) | / | C, HCQ, LMWH, Toc | No | Hospitalized (D15) | Clinically recovered, discharged home - D21 |
| B4 | M | 56 | Malaise, dyspnoea, cough, fever, anosmia | CXR: Pneumonia - Billateral | / | / | C, HCQ, LMWH, L/R, Toc | No | Hospitalized (D06) | Clinically recovered, discharged home - D11 |
| B5 | F | 66 | Malaise, fever | CXR: Pneumonia - Billateral | H, Asthma | / | C, HCQ, LMWH, Toc | No | Hospitalized (D15) | Clinically recovered, discharged home - D20 |
| B6 | F | 62 | Malaise, dyspnoea, cough, fever | CXR: Pneumonia - Billateral | DBT, H | Bilateral pleural effusion | C, Ciprofloxacin, Hemomycin, Van, HCQ, LMWH | Yes - IV | Hospitalized (D06) | Exitus lethalis - D09 (ICU) |
| B7 | M | 58 | Malaise, dyspnoea, fever | CXR, CT: Pneumonia - Billateral | DBT, H, Stent, COPD | / | C, Van, Mem, HCQ, LMWH | Yes - NIV | Hospitalized (D31) | Clinically recovered, discharged home - D55 |
| B8 | M | 84 | Malaise, dyspnoea, fever | CXR: Pneumonia - Billateral | CVD (SOAS) | Pulmonary edema | C, P/T, HCQ, LMWH | Yes - IV | Hospitalized (D08) | Exitus lethalis - D16 (ICU) |
| B9 | F | 78 | Malaise, dyspnoea, fever | CXR: Pneumonia - Billateral | H, COPD | Left pneumothorax, Epileptic seizure | Van, Mem, LMWH | Yes - IV | Hospitalized (D08) | Exitus lethalis - D19 (ICU) |
| B10 | M | 76 | Malaise, dyspnoea, fever, cough | CXR: Pneumonia - Billateral | H, COPD | / | Piperacillin-tazobactam, Van, Cst, LMWH | Yes - IV | Hospitalized (D10) | Exitus lethalis - D37 (ICU) |
| B11 | M | 57 | Malaise, dyspnoea, fever, nausea | CXR: Pneumonia - Billateral | DBT, CVD (IM), CBV (CVI) | C. Difficile | C, Hemomycin, Ertapenem, Van, Mem, Met, Cst, HCQ, LMWH | Yes - IV | Hospitalized (D16) | Clinically recovered, discharged home - D40 |
| B12 | F | 57 | Malaise, dyspnoea, fever, cough | CXR: Pneumonia - Billateral | Epileptic seizures | / | Mem, Cst, HCQ, LMWH | Yes - IV | Hospitalized (D13) | Exitus lethalis - D40 (ICU) |
| B13 | F | 47 | Malaise, dyspnoea, fever | CXR: Pneumonia - Billateral | DBT, H, Obesitas | / | Tigecycline, Van, HCQ, LMWH, Toc | Yes - IV | Hospitalized (D11) | Exitus lethalis - D23 (ICU) |
| B14 | F | 70 | Malaise, fever | CXR: Pneumonia - Billateral | H, CBV (CVI) | / | Ciprofloxacin, Met, HCQ, LMHW, Oseltamivir | No | Hospitalized (D03) | Transfered to another clinic - D05 |
| B15 | M | 71 | Malaise, fever | CXR: Pneumonia - Billateral | H, Mb. Alzheimer | / | LMWH | Yes | Hospitalized (D12) | Exitus lethalis - D18 (ICU) |
| B16 | M | 67 | Malaise, dyspnoea, fever, cough | CXR: Pneumonia - Billateral | / | / | Mem, Van, HCQ, LWMH | No | Hospitalized (D08) | Clinically recovered, discharged home - D19 |
| B17 | M | 62 | Malaise, fever, cough | CXR: Pneumonia - Billateral | / | / | Mem, Van, HCQ, LWMH, Toc | Yes - NIV | Hospitalized (D05) | Clinically recovered, discharged home - D16 |
| B18 | F | 61 | Malaise, dyspnoea, fever, cough | CXR: Pneumonia - Billateral | / | / | Cefixime, HCQ, LMWH | No | Hospitalized (D06) | Clinically recovered, discharged home - D11 |
| B19 | M | 70 | Malaise, dyspnoea, fever | CXR: Pneumonia - Billateral | CBV (CVI x2), CVD (IM x2) | / | Ertapenem, Van, Met, Amikacin, HCQ, LMWH | Yes - IV | Hospitalized (D08) | Exitus lethalis - D14 (ICU) |
| B20 | F | 73 | Malaise, dyspnoea, fever, cough | CXR: Pneumonia - Billateral | H, DBT, AF | / | C, LMWH | No | Hospitalized (D06) | Clinically recovered, discharged home - D12 |
| AF: atrial fibrillation; C: ceftriaxone; CBV: cerebrovascular disease; CVI: chronic venous insufficiency COPD: chronic obstructive pulmonary disease; CT: computed tomography; CVD: cardiovascular disease; IM: DVT: deep vein thrombosis; CXR: chest Xray; DBT: diabetes; DYS: dyslipidemia; H: hypertension; HCQ: hydroxychloroquine; IV: invasive ventilation; ICU: intensive care unit; LMWH: low molecular-weight heparin; NIV: non-invasive ventilation; S: active smoker; US: thoracic ultrasound; Van-Vancomycin; L/R- lopinavir/ritonavir; Mem-Meropenem; Met-Metronidazole; Cst-Colistin; Toc-Tocilizumab | | | | | | | | | | |
