# Supplementary material for: Reduced Expression of Autophagy Markers and Expansion of Myeloid-Derived Suppressor Cells Correlate With Poor T Cell Response in Severe COVID-19 Patients
Source: Front Immunol. 2021 Feb 22;12:614599. doi: 10.3389/fimmu.2021.614599 (PMC7937809; doi:10.3389/fimmu.2021.614599)
Supplement: Supplementary file 6 [file Table_4.docx]

| **Supplementary Table 4. Baseline Clinical and Laboratory Characteristics of Healthy donors and COVID-19 patients** | | | | |
| --- | --- | --- | --- | --- |
|  | **Healthy** | **Mild clinical course** | **Severe clinical course** | **p-value** |
| Number of patients | 16 | 21 | 20 |  |
| Age (Years, mean±SD) | 52.4 ± 15.7 | 53.4 ± 16.8 | 63.1 ± 11.8 | *>0.999  Δ 0.0868  # 0.1557 |
| Male gender (n,%)  Female gender (n,%) | 8 (50%) | 12 (57%) | 12 (60%) |  |
|  | 8 (50%) | 9 (43%) | 8 (40%) |  |
| **Laboratory values** | | | | |
| Total white blood cells count (x10P^9^P/L) | 6.3 ± 1.6 | 6.9 ± 3.1 | 9.4 ± 4.5 P^Δ, #^ | *0.248  Δ 0.007  # 0.022 |
| Neutrophils(x10P^9^P/L) | 3.9 ± 1.2 | 4.5 ± 2.6 | 7.7 ± 4.5P ^Δ, #^ | *0.238  Δ 0.001  # 0.004 |
| Lymphocytes (x10P^9^P/L) | 2.2 ± 0.7 | 1.8 ± 0.7 | 1.0 ± 0.7P ^ΔΔΔ, #^ | *0.121  Δ 0.00002  # 0.001 |
| Monocytes (x10P^9^P/L) | 0.2 ± 0.07 | 0.5 ± 0.2*** | 0.5 ± 0.3P ^ΔΔΔ^ | *0.00001  Δ 0.00004  # 0.344 |
| Red blood cells (x10P^12^P/L) | 4.4 ± 1.1 | 4.2 ± 0.9 | 4.1 ± 0.7 | *0.319  Δ 0.160  # 0.286 |
| Hemoglobin (g/L) | 144.6 ±11.0 | 123.1 ± 26.7* | 121.7 ± 22.4P ^ΔΔΔ^ | *0.004  Δ 0.0004  # 0.337 |
| Thrombocytes (x10P^9^P/L) | 269.3 ± 43.1 | 243.4 ± 112.3 | 241.3 ± 119.8 | *0.298  Δ 0.191  # 0.355 |
| D-dimer (ng/ml) | 115.2 ± 54.1 | 708.1 ± 1015.3*** | 1060.4 ± 762.2P ^ΔΔΔ^ | *0.00005  Δ 0.00002  # 0.112 |
| aPTT (sec) | 10.6 ± 0.7 | 12.6 ± 1.2*** | 17.9 ± 10.3P ^Δ, #^ | *0.00002  Δ 0.007  # 0.017 |
| Fibrinogen (g/L) | 3.5 ± 0.7 | 4.5 ± 1.2* | 4.7 ± 1.4P ^Δ^ | *0.003  Δ 0.003  # 0.354 |
| INR | 2.8 ± 0.4 | 1.1 ± 0.1*** | 1.6 ± 0.9P ^ΔΔΔ, ##^ | *0.00001  Δ 0.00004  # 0.0003 |
| CRP (mg/L) | 1.4 ± 1.1 | 13.0 ± 22.7* | 103.8 ± 105.6P ^ΔΔ, #^ | *0.025  Δ 0.0002  # 0.029 |
| Ferritin (ug/L) | 57.6 ± 40.9 | 272.0 ± 345.8* | 1957.5 ±  2693.0 P^Δ,#^ | *0.01  Δ 0.004  # 0.005 |
| AST (U/L) | 21.0 ± 5.9 | 27.6 ± 16.7 | 59.8 ± 53.0P ^Δ, #^ | *0.071  Δ 0.004  # 0.007 |
| ALT (U/L) | 22.2 ± 8.8 | 34.6 ± 32.1 | 87.2 ± 100.7P ^Δ, #^ | *0.071  Δ 0.008  # 0.014 |
| gGT (U/L) | 26.3 ± 5.8 | 43.3 ± 66.6 | 106.8 ± 104.7P ^Δ, #^ | *0.154  Δ 0.002  # 0.024 |
| LDH (U/L) | 255.0 ± 85.5 | 461.8 ± 103.6** | 969.6 ± 764.1P ^Δ, #^ | *0.0001  Δ 0.002  # 0.006 |
| Urea (mmol/L) | 5.5 ± 1.4 | - 1. ± 5.3 | 11.5 ± 10.1P ^Δ, #^ | *0.198  Δ 0.013  # 0.004 |
| Comparison of the respective groups by the Student t-test: *p<0.05, **p<0.001, ***p<0.0001; Δ p<0.05, ΔΔ p<0.001; ΔΔΔ p<0.0001; # p<0.05, ## p<0.001, ###p<0.0001. Statistical significance between: healthy volunteers vs. patients with mild clinical course (*), healthy volunteers vs patients with severe clinical course (Δ), patients with mild vs. severe clinical course (#). | | | | |
